# Pregnancy in patients with chronic kidney disease undergoing dialysis

**DOI:** 10.1590/2175-8239-JBN-2024-0067en

**Published:** 2024-11-08

**Authors:** Fernanda Salomão Gorayeb-Polacchini, Ana Flavia Moura, Claudio Luders, José Andrade Moura, Juliana El Ghoz Leme, Dirceu Reis da Silva

**Affiliations:** 1 Sociedade Brasileira de Nefrologia, São Paulo, SP, Brazil.; 2 Hospital de Base de São José do Rio Preto, São José do Rio Preto, SP, Brazil.; 3 Escola Bahiana de Medicina e Saúde Pública, Salvador, BA, Brazil.; 4 Hospital Sírio-Libanês, São Paulo, SP, Brazil.; 5 Nefroclínicas Curitiba, Curitiba, PR, Brazil.; 6 Hospital de Clínicas de Porto Alegre, Porto Alegre, RS, Brazil.

**Keywords:** Chronic Kidney Disease, Dialysis, Pregnancy, Pregnancy Complications, Preeclampsia

## Abstract

Women with chronic kidney disease are less likely to become pregnant and are more susceptible to pregnancy complications when compared to patients with normal kidney function. As a result, these are considered high-risk pregnancies, both maternal and fetal. Over the years, there has been an increase in the incidence of pregnancies in dialysis patients, and an improvement in maternal and fetal outcomes. It is believed that the optimization of obstetric and neonatal care, the adjustment of dialysis treatment (particularly the increase in the number of hours and weekly frequency of dialysis sessions), and the use of erythropoiesis-stimulating agents have provided better metabolic, volume, blood pressure, electrolyte, and anemia control. This review article aims to analyze pregnancy outcomes in chronic kidney disease patients undergoing dialysis and to review nephrological medical management in this scenario. Due to the growing interest in the subject, clinical recommendations for care practice have become more consistent in both drug and dialysis management, aspects that are addressed in this review.

## Introduction

Chronic kidney disease (CKD) affects approximately 3% of women of reproductive age and is a factor for high-risk pregnancy. With the decrease in glomerular filtration rate, there is a progressive reduction in fertility and an increased risk of unfavorable pregnancy outcomes. Few studies have explored the pathophysiological mechanisms underlying this association^1,2^. Hyperprolactinemia and low serum estradiol levels observed in dialysis patients are linked to alterations in gonadotropin-releasing hormone and the hypothalamic-pituitary-gonadal axis, and may account for ovulation difficulties. There are still conflicting results associating low serum anti-Mullerian hormone levels observed in dialysis patients with impaired fertility^3,4^.

## Discussion

### Epidemiology

Over the years, interest in fertility and pregnancy in dialysis patients has increased, enabling a progressive improvement in maternal and fetal prognosis. Table 1 shows the evolution of the increasing incidence of pregnancy rates in this population^5,6^. An American cohort study has demonstrated that caucasian women on dialysis have a higher incidence of pregnancy, with diabetes mellitus (DM), peritoneal dialysis (PD) modality, and longer dialysis vintage being additional characteristics that contribute to reduced fertility^6^. Pregnancies initiated in chronic kidney disease patients without dialysis treatment have a higher rate of live births (91%) compared to those initiated during the dialysis phase (66%)^7^.

**Table 1:** Incidence of pregnancies on dialysis over the years

Study	Year	Incidence
ANZDATA^5^	1976–1985	0.54 per 1,000 patient-years
	1986–1995	0.67 per 1,000 patient-years
	1996–2008	3.3 per 1,000 patient-years
Shah et al.^6^	2005–2013	17.8 per 1,000 patient-years
		40.9 per 1,000 patient-years (patients aged 20–24)

Several aspects seem to explain the increase in incidence and better pregnancy outcomes over the years: 1) improved management of anemia with erythropoiesis-stimulating agents (ESAs), 2) improvement in hemodialysis treatment with biocompatible membranes, 3) increased weekly dialysis dose, through higher frequency or longer sessions, providing lower exposure to the uremic environment and better volume and blood pressure control, and 4) advances in obstetric and neonatal monitoring and treatment^1,2,5,8,9,10^.

### Pregnancy-Related Outcomes and Complications

A systematic review of 14 studies conducted between 2010 and 2020, involving 11 countries (from 6 continents), and including 2,754 pregnancies in 2,364 dialysis patients, provided comprehensive data on the scenario of pregnancy in dialysis over the past decade (Table 2)^11^. The primary modality of renal replacement therapy was hemodialysis (HD), in 92.6% of cases^11^. Unlike older reviews^5,6,12,13^, this study included a larger number of pregnancies and showed a better prognosis^11^.

**Table 2:** Pregnancy outcomes and complications in women on dialysis^11^

Outcomes and complications	Results
**Pregnancy-related outcomes**	
Gestational age at birth	25 to 36 weeks
Cesarean section	9% to 100%
Spontaneous abortion	16.9%
Stillbirth	8.3%
Polyhydramnios	17.7%
Pregnancy termination	5.2%
**Causes of pregnancy termination**	
Hypertensive disorders of pregnancy	17.1%
Preterm labor	8.1%
Intrauterine growth restriction	6.5%
Premature placental abruption	3.2%
Cervical insufficiency	2.3%
Fetal distress	2.3%
Prior cesarean section	2.3%
**Maternal complications**	
Hypertension	7.7%
Preeclampsia	11.9%
Eclampsia	0.7%
HELLP Syndrome	0.7%
Anemia	3.9%
Gestational diabetes	0.5%
Peritonitis	0.2%
Cholestasis	0.7%
Maternal death*	0%

HELLP: hemolysis, elevated liver enzymes, and low platelet counts.

*From pregnancy to 42 days postpartum (WHO).

A complication to monitor in this population is polyhydramnios, which can affect up to 53% of pregnant women in some studies. This complication may indicate the need to increase the dialysis dose and vintage, as well as adjust the dry weight^14^.

## Maternal Complications During Pregnancy

Table 2 also lists the major maternal complications in pregnant women undergoing dialysis^11^, with particular emphasis on hypertensive syndromes. Over the past few years, there has been a reduction in the incidence of severe forms of these syndromes, due to more intensive dialysis and improved obstetric monitoring^11,12,15,16^.

Similarly to patients who are not on dialysis treatment, there is a higher risk of maternal and fetal complications in dialysis patients who are primigravida, older, obese, and those with a prior diagnosis of arterial hypertension (AH), systemic lupus erythematosus, and DM^17,18^.

## Hypertensive Syndromes of Pregnancy

Hypertensive syndromes are the most common cause of death among pregnant women, as they elevate maternal cardiovascular (CV) risk, including after pregnancy^15,16,19^. Recommendations for the management of these conditions for pregnant women on dialysis are largely derived from those for pregnant women not on dialysis, with adjustments made to the particularities of CKD.

### Chronic Hypertension (CH)

Chronic hypertension (CH) is defined as an elevation in systolic blood pressure (SBP) ≥ 140 mmHg or diastolic blood pressure (DBP) ≥ 90 mmHg, measured on at least two occasions at a minimum interval of 4 hours, before the 20th week of pregnancy, or persisting for 12 weeks or more after delivery. In patients with severely elevated blood pressure (SBP ≥ 160 mmHg or DBP ≥ 110 mmHg), a single measurement can be used to diagnose CH, thereby facilitating the initiation of therapy^20,21^. To date, there is no consensus regarding the optimal blood pressure target for hypertensive pregnant women. Different targets have been proposed, and it seems reasonable to aim for SBP < 140 mmHg and DBP < 90 mmHg for hypertensive pregnant women on dialysis.

CV risk and mortality in hypertensive pregnant women are higher when compared to normotensive pregnant women, and blood pressure control reduces this risk^22^. In addition, there is an increased prevalence of gestational diabetes among women with hypertension compared to normotensive women, which seems to be attributable to shared risk factors^23^.

In order to mitigate obstetric risks, hypertensive women need to undergo assessment and adopt measures for blood pressure control while still in the pre-conception phase. Conditions such as obesity and DM, as well as lifestyle modifications, should be addressed and controlled prior to pregnancy. Furthermore, the antihypertensive regimen should be reviewed, with special attention to medications that are contraindicated or have limited evidence of safety during pregnancy^20^. The antihypertensive drugs available in Brazil that are recommended for use in pregnant women are: metoprolol or pindolol, long-acting nifedipine, methyldopa, and hydralazine.

Beta-blockers are the most commonly used antihypertensive drugs during pregnancy. Although some studies have suggested associations between this class of drugs and restricted placental blood flow, intrauterine growth restriction, fetal weight restriction, fetal malformations, neonatal hypoglycemia, and increased perinatal mortality (as observed with atenolol, for instance), the majority of studies have not demonstrated an excess risk of unfavorable obstetric outcomes compared to other antihypertensive drugs approved for use during pregnancy^24,25^. The use of labetalol (not available in Brazil), pindolol, or metoprolol is particularly recommended. Propranolol shows conflicting results across studies regarding neonatal complications. Nevertheless, the drug may be used in lactation^26^.

Calcium channel blockers are also widely used in hypertensive pregnant women, especially in the second half of pregnancy, due to their tocolytic effects. Among them, nifedipine (extended- or intermediate- release presentations) is the most widely used^27^. Despite being widely used in the general hypertensive population, amlodipine is relatively little used in pregnant women due to the paucity of data in literature, although there is evidence of safety in a study involving 48 patients^28^. Non-dihydropyridines are also effective during pregnancy; however, caution is required as they are associated with interference in fetal heart rate^29^.

Methyldopa is the antihypertensive drug with the longest history of use during pregnancy, and there is extensive knowledge regarding its potential adverse effects. Methyldopa has been demonstrated to have a lower risk than other antihypertensives approved for use during pregnancy, also when compared to labetalol^30^. The disadvantages of methyldopa include its delayed onset of action (3–6 hours) and its low potency, often requiring high doses (increased risk of hepatotoxicity, hemolytic anemia, and sedative effects). Additionally, its moderate dialyzability and short dosing interval limit its use as an optimal drug for pregnancy on dialysis. Overall, it is preferable to use methyldopa as part of a combination therapy^20,25^.

Hydralazine can be used during pregnancy both orally and intravenously. Its primary indication is for the acute treatment of hypertensive crises, due to its rapid onset of action (10–20 minutes)^31^. In these cases, the incidence of adverse effects is higher, affecting approximately 50% of patients^20^. Particular attention should be paid to hypotension, which increases maternal and fetal risk due to reduced uteroplacental blood flow. Intermittent intravenous infusion of 5–10 mg of hydralazine every 20–40 minutes is then recommended, not exceeding the maximum dose of 20 mg^32^. In maintenance regimens, hydralazine is more commonly used in combination therapies, especially with beta-blockers, which reduce some of its adverse effects.

Thiazide or loop diuretics are considered safe during pregnancy, despite concerns about their effects on maternal blood volume and the risk of reduced uteroplacental perfusion, especially in the first two weeks of use, when plasma volume reduction is greater^20,33^. In pregnant women with CKD, its use is generally safe, given the role of hypervolemia in AH. In the context of pregnancy in women undergoing dialysis treatment, intensive dialysis reduces the need for diuretics, which is usually reserved for pregnant women with heart failure or non-dialysis CKD. In refractory cases of hypervolemia, a combination of diuretics could be used^33^.

Renin-angiotensin-aldosterone system inhibitors (RAASi) are strongly associated with renal abnormalities in the fetus when used during the second half of pregnancy^34^. Although the risks associated with RAASi during the first trimester are not well defined, it is recommended that they be replaced either during the preconception period or as soon as pregnancy is diagnosed^34^.

In general, mineralocorticoid receptor antagonists are not recommended during pregnancy, primarily due to the scarcity of data in the literature and concerns regarding antiandrogenic effects on fetal development. Novel drugs in this class have a reduced antiandrogenic effect, but there is limited experience with their use in pregnant women^35^.

Sodium nitroprusside may be used in specific situations, such as antepartum hypertensive emergencies, always for short periods, carefully considering the risk-benefit ratio, due to inconclusive evidence on the risk during pregnancy^36^.

### Gestational Hypertension

Gestational hypertension consists of the onset of AH occurring at 20 weeks of pregnancy or more, with no signs or symptoms that would suggest preeclampsia (PE)^15^. If the patient continues to present with elevated BP for 12 weeks or more after delivery, the diagnosis of gestational hypertension should be replaced by that of CH.

Generally, gestational hypertension is classified into severe (SBP ≥ 160 mmHg or DBP ≥ 110 mmHg) and non-severe (SBP < 160 mmHg or DBP < 110 mmHg.) Severe gestational hypertension is associated with greater maternal and fetal morbidity and an increased risk for the mother of developing CH^37^.

Among pregnant women with gestational hypertension, 10–50% will develop PE between 1–5 weeks after diagnosis^38^, with uncertainty as to whether or not this represents an early stage of PE. Severe gestational hypertension and PE should be managed in a similar way, with immediate initiation or adjustment of the antihypertensive regimen and inpatient monitoring^39^.

### Preeclampsia

PE is defined as AH beginning after the 20th week of pregnancy, associated with proteinuria (≥300 mg/24 hours or urinary protein/creatinine ratio ≥0.3 mg/mg) or target organ damage such as thrombocytopenia (<150,000/mm^3^), liver dysfunction (AST or ALT transaminases > 40 IU/L), kidney injury (creatinine > 1 mg/dL), or pulmonary edema. Additionally, signs of placental insufficiency (such as fetal growth restriction and/or fetal Doppler velocimetry changes) should also draw attention to the diagnosis of PE, even in the absence of proteinuria. It is noteworthy that PE can appear during pregnancy or even after childbirth, although it is rare after the second day postpartum^40^.

The diagnosis of PE is particularly challenging in pregnant women on dialysis considering the high prevalence of CH and previous increases in creatinine and proteinuria. It is necessary to monitor the development of extrarenal manifestations (hematological, hepatic, and neurological alterations) in addition to associated fetal manifestations^41^. There is a lack of more accurate diagnostic resources, including the use of biomarkers such as angiogenic factors (e.g. the sFlt-1/PlGF ratio), which could assist in the management of doubtful cases. This is particularly useful in order to exclude the diagnosis in mild and moderate cases, thereby avoiding unnecessary interventions for some pregnant women^42^.

PE may be classified according to gestational age at diagnosis: early-onset (<34 weeks, associated with a worse prognosis) or late-onset (≥34 weeks)^41^.

In women with preexisting CH, superimposed PE is characterized by: 1) increased blood pressure levels, requiring adjustment of the antihypertensive regimen, 2) increased proteinuria already detected in the first half of pregnancy, 3) occurrence of target organ damage, or 4) presence of signs of placental insufficiency, such as fetal growth restriction and/or fetal Doppler velocimetry changes^21,41^.

Formerly referred to as severe PE, “PE with severe features” (or clinical and/or laboratory deterioration) is defined by the presence of: 1) hypertensive crisis with BP ≥ 160 and/or 110 mmHg persistent after 15 minutes; 2) hypertensive emergency; 3) impending eclampsia; 4) eclampsia; 5) HELLP syndrome (hemolysis, elevated liver enzymes, and low platelet count); 6) acute kidney injury with creatinine ≥ 1.2 mg/dL or oliguria < 500 mL/24h; 7) chest pain; or 8) pulmonary edema^40^.

The presence of PE in pregnant women on dialysis increases the risk of an unfavorable fetal outcome over 20-fold, and is the main determinant of evolution^18^. Therefore, prevention and diagnosis strategies, along with specific follow-up protocols, conducted by experienced multidisciplinary teams, are paramount for an appropriate fetal outcome^43,44^.

The benefit of using acetylsalicylic acid to prevent PE in pregnant women is well documented; for this reason, it should be used in pregnant women undergoing dialysis, despite the lack of studies in this population, provided there are no specific contraindications to its use. Recommended doses range between 75–162 mg a day (100 mg being the most commonly used in Brazil). It is recommended that this prophylaxis be initiated between the 12th and 16th weeks of pregnancy, or up to the 28th week in cases of late diagnosis^45^. The use of low doses of acetylsalicylic acid has not been associated with a higher rate of childbirth complications, suggesting that it could be maintained until delivery or discontinued 5–10 days prior to it^45^.

Another recommended measure is calcium supplementation, especially in populations with low calcium intake (such as the Brazilian population), starting in the first trimester and maintaining it until the end of pregnancy, at the recommended dose of calcium carbonate 1-2 g/day, or calcium citrate 2–4 g/day, divided into 3 doses^40^. Better absorption is achieved when administered in small doses (500 mg), especially during meals. Calcium Citrate, however, may be taken between meals, which is the recommended form for patients with absorption disorders^46^.

All pregnant women with PE with severe features or with difficult-to-control hypertension, even if asymptomatic, should be prescribed magnesium sulfate before delivery in order to prevent maternal seizures (eclampsia)^40^. Given that magnesium is excreted through the kidneys, there is a risk of potentially fatal toxicity (respiratory or central nervous system depression, and arrhythmias) in pregnant women undergoing dialysis. Therefore, in such cases, half the recommended dose should be administered and the infusion rate reduced, with careful monitoring of serum magnesium levels and clinical signs of toxicity (respiratory depression and abolished tendon reflex). If intoxication is suspected, the need for administering intravenous calcium gluconate to antagonize the deleterious effects of magnesium should be evaluated^45^.

By definition, changes associated with PE evolve towards resolution after childbirth.

### Eclampsia

Eclampsia is defined as the unexplained occurrence of convulsive seizures or coma in pregnant women with hypertensive disorders of pregnancy. There may be precursor signs and symptoms of convulsive seizures (such as severe headache, visual changes, upper abdominal pain and hypertensive spike)^45^. With a decreasing prevalence in developed countries, it remains common in some developing countries. Eclampsia is the primary complication of preeclampsia, and occurs in 2-3% of pregnant women with PE with severe features, particularly in those under 20 or over 35 years of age^47^. Although 60% of eclampsia cases manifest during pregnancy, some develop intrapartum or in the postpartum period (90% of these cases occurring within the first week postpartum)^44^.

Generalized tonic-clonic seizures, with a sudden onset and no identified cause, are the most typical presentation, with focal, multifocal seizures, or even coma being less common^45^. In pregnant women with generalized tonic-clonic seizures who progress without neurological deficits, additional diagnostic evaluation is not required, which should be reserved for atypical cases (pregnant women without diagnosed hypertensive syndromes or with neurological deficits)^48^.

Magnesium sulfate is the drug of choice for the prevention and treatment of eclampsia, and is also indicated in the presence of prodromes^40^. In asymptomatic patients, there is no consensus on the prophylactic use of magnesium sulfate, although prophylaxis is advocated in all pregnant women with PE, considering the low toxicity in the general population^49^. Therefore, its use should also be considered in pregnant women on dialysis, with appropriate care due to the increased risk of magnesium intoxication^45^.

The treatment of convulsive seizures should be associated with AH control and preserve the maternal-fetal binomial. Measures to be adopted include ensuring maternal oxygen administration (measuring oximetry, using supplemental oxygen), correcting acidosis, and minimizing the risk of bronchoaspiration (positioning the pregnant woman in the lateral decubitus position, aspirating secretions, and inserting a mouth guard) during the seizure. In cases where BP is ≥ 160/110 mmHg, the preferred treatment should be intravenous hydralazine or oral nifedipine. Sodium nitroprusside may be used, particularly for pulmonary edema associated with heart disease, for a short period (6–12 hours), considering the poorly documented risk of fetal neurological toxicity^31,40,49^.

### HELLP Syndrome

In general, HELLP syndrome occurs between the 28th and 36th weeks of pregnancy, and the most common clinical symptom is upper quadrant abdominal pain, mainly in the epigastrium. It comprises the presence of 1) hemolysis, identified by lactate dehydrogenase (LDH) twice or more the upper limit of normal, serum indirect bilirubin ≥ 1.2 mg/dL, haptoglobin < 25 mg/dL, or the presence of schistocytes on a peripheral blood smear; 2) hepatic failure, indicated by transaminases levels (AST and/or ALT) > 70 IU/L; and 3) thrombocytopenia (<100,000 mm^3^)^40^.

Associated with a high risk of obstetric complications, HELLP syndrome should be managed in a hospital setting, with immediate treatment of severe AH. Hepatic hemorrhage is common and should be considered in patients with severe upper quadrant abdominal pain. If confirmed, the pregnancy should be terminated by caesarean section as soon as hemodynamic stability is achieved. To prevent maternal seizures and provide fetal neuroprotection, magnesium sulfate should be initiated early and maintained for 24–48 hours postpartum^50^.

### Obstetric Management in Preeclampsia

In women with PE at a gestational age of under 23 weeks, expectant management is associated with high perinatal mortality and maternal morbidity and mortality. Therefore, in cases of PE with severe features (clinical and/or laboratory deterioration), it is recommended to terminate the pregnancy, as neonatal viability is low. It is imperative that this decision be made in consultation with the family^51^.

In PE cases with a gestational age between 23–34 weeks, considering the burden of prematurity, care in a specialized center is suggested, aiming at preserving the fetus. In PE with severe features, after maternal stabilization^39^, it is recommended that the pregnancy be terminated at 34 weeks, considering the increased maternal and fetal morbidity following this gestational age^39^. In the absence of severity signs of PE, with a gestational age between 34–37 weeks, it is acceptable to defer termination of pregnancy until the 37th week, in a specialized center^40,53^.

If ensuring maternal and fetal well-being is not possible, termination of pregnancy should be indicated, regardless of gestational age^45^. The criteria for termination are: 1) HELLP syndrome; 2) impending eclampsia refractory to treatment; 3) eclampsia; 4) placental abruption; 5) AH refractory to treatment with three antihypertensive drugs; 6) pulmonary edema/cardiac involvement; 7) progressive laboratory changes; 8) progressive elevation of urea and creatinine, oliguria, and anasarca; 9) hepatic hematoma or rupture; and 10) fetal vitality changes^52^.

## Urinary Tract Infection in Pregnancy

Common during pregnancy, urinary tract infection (UTI) may present as cystitis or pyelonephritis, the latter being more common in pregnant women due to smooth muscle relaxation with subsequent dilatation of the ureter and the compressive effect by the gravid uterus on the bladder and ureter^54^. When residual diuresis is present in pregnant women on dialysis, the clinical presentation is similar to that of women not on dialysis, while in anuric pregnant women only abdominal pain and fever may occur^55^.

More frequent in the first trimester of pregnancy, asymptomatic bacteriuria occurs in 2–7% of pregnant women^54^, and may evolve to symptomatic conditions in up to 35% of women if left untreated. Women with a history of UTI, prior abortion^56^, with DM or autosomal dominant polycystic kidney disease, and those of low socioeconomic status are particularly at risk^54,57^. Screening with urine culture is recommended at the first prenatal visit or during the first trimester. However, the frequency of further evaluations is controversial; the Italian positioning recommends monthly collection for high-risk patients^56^. Treatment seems to be associated with a reduced risk of pyelonephritis, prematurity, low birth weight, perinatal mortality^57^, and PE^58^, although not all studies demonstrate the same association^54,59^.

Present in up to 2% of pregnancies, pyelonephritis is more frequent in the second and third trimesters and may be associated with maternal morbidity and preterm delivery^60^. Considering the maternal and fetal risks, and the greater association with structural abnormalities of the urinary tract^54^, it is recommended that pregnant women on dialysis with pyelonephritis should be treated in a hospital setting with intravenous antibiotics. Additionally, they should undergo ultrasound of the urinary tract when there is a history of previous pyelonephritis, renal lithiasis, immunosuppression, and urological disease^56^.

Cystitis in pregnant women undergoing dialysis is managed with antibiotics administration, preferably beta-lactams, first or second-generation cephalosporins, or fosfomycin. The use of third-generation cephalosporins, piperacillin-tazobactam, or carbapenems is reserved for cases of pyelonephritis. Aminoglycoside antibiotics, fluoroquinolones, nitrofurantoin, trimethoprim-sulfamethoxazole, and tetracycline should be avoided in pregnant women on dialysis due to their association with teratogenicity or fetal toxicity^56,61^.

## Neonatal Outcomes and Complications

Major neonatal complications in pregnancies of dialysis patients include abortion, fetal death, preterm birth, and low birth weight^11^.

The live birth rate has increased due to the greater availability of dialysis hours and days, as well as improved prenatal and neonatal care^7,11^. There remains a significant difference between countries, and even between dialysis services regarding live birth rates and preterm births^11^. This is presumably due to the diversity in dialysis practices^11,13^, population differences, and disparities in obstetric care^9^. It should be added that the live birth rate is higher in pregnancies of women with pre-dialysis CKD, and even in those undergoing dialysis with higher residual renal function^7^. Table 3 lists the frequencies of the main neonatal complications^11^.

**Table 3:** Neonatal outcomes and complications in pregnancies of dialysis patients^11^

Neonatal complications and outcomes	Results
Live births	71.4%
Birth weight	590 to 3500 g
Preterm birth	82.8%
Neonatal ICU requirement	65.5%
Small for gestational age	32%
ARDS	2.4%
Neonatal deaths*	7.6%
Perinatal deaths^#^	15.3%

ICU: intensive care unit, ARDS: acute respiratory distress syndrome.

*Neonatal deaths: deaths between the 1st and 28th day after birth.

#Perinatal deaths: deaths from 22 complete weeks of pregnancy to the 6th full day of life.

A study of pregnancy in dialysis patients found a 0.3% rate of fetal malformations, suggesting that these are not more frequent in pregnant women under dialysis. Findings include: duodenal atresia, cleft palate, ear abnormalities, abdominal hernia, intellectual disability, retinopathy, glaucoma, pyelocaliceal dilatation, among others^11^.

## Non-Dialysis Care During Pregnancy

### Preconception Counseling

Every dialysis patient of childbearing age should be informed about the prospects of pregnancy in this context, including maternal and fetal prognosis. They should also be advised on contraceptive strategies. Proper management of chronic diseases, such as AH, DM, and autoimmune diseases, is essential to promote a better outcome.

In women of childbearing age who wish to conceive, with no advanced maternal age, and no contraindications to transplantation, it is recommended to wait until kidney transplantation is performed. This is due to better pregnancy outcomes with a functioning graft^62^. Fertility is at least partially restored after kidney transplantation, with the best chance of a successful pregnancy being associated with younger maternal age, normotension, no obesity or proteinuria, at least two years after transplantation, normal kidney function, and no previous episodes of rejection. This ideal profile may not be fully required for patients who need to wait for a deceased donor graft, as the time spent on the waiting list could impact decisions regarding conception planning. Even under ideal conditions, the risk of complications remains higher than in the general population^62^.

Patients on dialysis or who have undergone kidney transplantation may choose assisted reproduction while recognizing the increased risk of bleeding, thrombosis, and infection in case of a twin pregnancy. It should be emphasized that ovarian hyperstimulation could lead to worsening renal function in patients with chronic nephropathy and increase the risk of hypertensive disorders of pregnancy^63^.

### Post-conception care

For diagnosing pregnancy in women on dialysis, the use of *beta*-HCG levels is limited, as they are usually increased in patients with advanced CKD, leading to possible false-positive results. Therefore, it is necessary to complement the assessment with an obstetric ultrasound examination^64^.

In the event of pregnancy: 1) the multidisciplinary team of the dialysis service should be notified; 2) advise increasing the length and frequency of dialysis sessions; 3) refer to high-risk prenatal care; 4) evaluate medications in use, discontinuing those with potential teratogenic effects; 5) check vaccination records; and 6) establish an individual clinical and laboratory monitoring plan^43^.

Obstetric follow-up also includes continuous monitoring of fetal growth and development, and screening for malformations, as well as placental conditions. The route of delivery should be determined by obstetric indication, and vaginal delivery is not contraindicated for women with kidney disease^65^.

It is recommended that the frequency of high-risk prenatal visits be (at least) once a month during the first trimester, every two weeks during the second trimester, and weekly during the third trimester. This increased frequency is due to significant changes in volume status and a higher risk of BP-related complications during the third trimester^65^.

Laboratory tests should be monitored regularly (at least) every two weeks, especially urea, bicarbonate, electrolytes, glucose, blood count, calcium, and phosphorus levels. After the 20th week, liver function tests and markers of hemolysis should be included, given the possible occurrence of HELLP syndrome^14^.

For all pregnant women without a prior diagnosis of DM, regardless of the presence of risk factors, it is recommended that the diagnostic investigation for gestational diabetes be performed between the 24th and 28th week of pregnancy, through an Oral Glucose Tolerance Test^66^.

Table 4 shows the main medications used in dialysis patients and the FDA (Food and Drug Administration) classification regarding pregnancy^67^.

**Table 4:** Main medications used in CKD patients during pregnancy^67^

Categories	Drugs	FDA categories
**Drugs considered safe**		
Erythropoiesis-stimulating agent	Erythropoietin	Not assigned
Alkali	Sodium bicarbonate	C
Antiplatelet agent	Acetylsalicylic acid	A
Anticoagulant	Low-molecular-weight heparin	B
Antihypertensives	Alpha-methyldopa	B
	Pindolol	B
	Hydrochlorothiazide	B
	Clonidine	C
	Metoprolol	C
	Nifedipine	C
Uricosuric agent	Allopurinol	C
Intravenous iron supplementation	Ferric hydroxide saccharate	B
Oral iron supplementation	Iron sulfate	Not assigned
Calcium supplementation	Calcium carbonate	Not assigned
Vitamin D	Cholecalciferol	Not assigned
**Unsafe drugs**		
Anticoagulant	Warfarin	Not assigned
	Rivaroxaban/Apixaban	C
Vitamin D analogs	Calcitriol/Paricalcitol	C
Antihypertensives	Atenolol	D
	Short-acting nifedipine	X
	Angiotensin-converting enzyme inhibitors	X
	Angiotensin receptor blockers	X
Calcimimetic	Cinacalcet	C
Phosphate binder	Sevelamer	C

FDA: Food and Drug Administration. Category A refers to evidence that there is no risk to fetuses in controlled human studies; Category B refers to the absence of evidence of risk to the fetus; Category C refers to the impossibility of excluding risk to the fetus; Category D refers to positive evidence of risk to the fetus; and Category X refers to contraindication during pregnancy.

### Diet and Nutritional Management

The nutritional plan for pregnant women undergoing dialysis should respect the increased need for energy, proteins, vitamins, and minerals during pregnancy, so as to promote fetal growth and development, while also addressing the specific issues related to CKD^68^.

It is recommended that caloric needs be calculated based on the requirement of 35 Kcal/kg/day (pre-pregnancy weight), plus the nutritional energy needs related to pregnancy, which are estimated at 85 Kcal/day in the first trimester, 285 Kcal/day in the second trimester, and 475 Kcal/day in the third trimester^69^. In pregnant women on PD, where there is absorption of existing dialysate glucose, the basal caloric intake should be 25 Kcal/kg/day plus specific gestational needs^70^. Of the total daily caloric intake, it is recommended that 30-35% come from fats, and 45-65% from carbohydrates^56^.

Regarding protein intake, 1.5–1.8 g/kg/day + 10 g/day is suggested, due to protein loss during intensified dialysis treatment in pregnancy^14^.

Usually, the increase in protein and caloric intake is accompanied by a progressive increase in dry weight, with an expected gain of 1–1.5 kg during the first trimester, and 0.5 kg/week starting from the second trimester^56^.

Deficiency in micronutrients (especially water-soluble and dialyzable vitamins and minerals) may impact fetal organogenesis. Supplementation with folic acid, vitamin C, thiamine, riboflavin, niacin, vitamin B6 and zinc is recommended^68^.

Sodium and potassium intake should be individualized, based on serum electrolyte concentrations, BP, medications, and volume status^68^.

### Treatment of Mineral and Bone Disorder

Among pregnant women on dialysis, calcium supplementation (via oral formulation or dialysis solution) is generally necessary for fetal bone formation and prevention of PE. Regarding calcium supplementation, a systematic review concluded that there was a 55% reduction in the risk of PE, which was even more pronounced in women with a low-calcium diet (74%) and those at high risk of PE (78%)^71^. Therefore, all women should be advised to follow a calcium-rich diet during pregnancy, with supplementation recommended for those at risk of PE and/or on a low-calcium diet^71^ (see section on Preeclampsia). Serum calcium levels should be monitored, avoiding hypo- or hypercalcemia (risk of fetal hypoparathyroidism)^25^.

It is expected that, under an intensive HD regimen, the pregnant patient tends to develop hypophosphatemia. Therefore, weekly monitoring of serum phosphorus levels is recommended for eventual supplementation (orally, intravenously, or in the dialysate solution), minimizing maternal and fetal risks (cardiac, neurological, and rhabdomyolysis)^25^. In the unusual event of hyperphosphatemia, the phosphate binders of choice are calcium carbonate and calcium citrate, considering the safety and increased demand for this element during pregnancy. Other phosphate binders, such as sevelamer, lanthanum carbonate, calcium acetate, and ferric citrate, lack proven safety in pregnancy^72^.

Regarding secondary hyperparathyroidism, the target parathyroid hormone level for pregnant dialysis patients should follow the same pattern as the dialysis population. However, it is important to consider the risks of managing this condition with drugs considered to be of limited safety (see Table 4)^25^. The risks of untreated hyperparathyroidism (uncommon due to intensified dialysis and increased calcium supply) could pose a risk to the fetus (hypocalcemia, secondary hyperparathyroidism, and rickets)^25^.

Pregnant women on dialysis should supplement with vitamin D3, usually at a dose of 800–1000 IU/day^70^. The optimal level of vitamin D for pregnant women on dialysis has not yet been established^73^. Nonetheless, the use of cholecalciferol (a safer formulation in pregnancy) is suggested to achieve serum levels above 30 ng/mL^25^.

### Treatment of Anemia

In addition to the usual factors of anemia (resulting from CKD and chronic HD^74^), there are further needs of the developing fetus and the disproportion between the growing increase in maternal plasma volume in relation to erythrocyte production^75^.

Iron supplementation is generally necessary, especially after the second trimester^76^ with intravenous administration being a more effective alternative, since enteral iron absorption is reduced in CKD^74^. The treatment of iron-deficiency anemia in pregnant women should initiate when the hemoglobin level is <11 g/dL^76^, and iron reserves should be adjusted in the usual way, according to current CKD guidelines. Ferritin levels should be maintained at >200 mg/dL for HD patients and >100 mg/dL for PD patients, with a transferrin saturation index of between 20–30%^74^.

Pregnancy-safe iron formulations are prioritized, with ferrous sulfate and ferric hydroxide saccharate being the most widely used forms in Brazil. Ferrous sulfate may be used during pregnancy (Category A)^77^, while ferric hydroxide saccharate may be employed on a risk-benefit basis. Although there are no studies in pregnant women, ferric hydroxide saccharate has shown no adverse fetal effects in animal studies (Category B)^76^. Iron sucrose has low allergenic potential, a lower tendency to accumulate in visceral organs, and has no toxic preservatives for pregnancy^25,78^. Conversely, sodium ferric gluconate contains preservatives with benzyl alcohol, which could lead to adverse fetal events^25^. Ferric carboxymaltose (despite a study showing safety^78^) and ferric derisomaltose have not been approved by the FDA for use in pregnant women^25^.

In the context of ESAs, most pregnant women on dialysis require them to maintain adequate hemoglobin levels (according to the current guidelines for anemia in CKD^74^), with dosage adjustments resulting in a 50–100% increase over the usual dose^79^. As with the rest of the dialysis population, using ESA doses to achieve higher-than-target hemoglobin increases the risk of AH and thrombosis^74^. Since ESAs do not cross the blood-placental barrier, they are considered safe during pregnancy, with epoetin alfa being the most extensively studied obstetric safety drug^79^. A study with darbepoetin alfa also demonstrated safety in pregnancy^79^.

Hypoxia-inducible factor prolyl hydroxylase enzyme inhibitors, oral medications for anemia in dialysis, have no proven safety for use in pregnant women undergoing dialysis^80^.

## Dialysis Treatment During Pregnancy

Currently, there is considerable evidence to support the idea that the highest dose of dialysis is crucial for a better fetal outcome. This is reflected in the recommendation that pregnant women should undergo dialysis at a frequency of 6-7 sessions per week, as increasing the weekly frequency of dialysis is essential for reducing maternal and fetal uremic environment^11,43,56^.

However, there is a significant heterogeneity among studies on the number of weekly hours and the monitoring of dialysis doses in pregnant patients^9,10,11,13,81,82,83^. The two study groups with the best outcomes regarding fetal survival employ quite different strategies. In Canada, Hladunewich et al.^82^ are based on the number of weekly hours of HD, with a recommended minimum dose of 36 hours per week. Meanwhile, Baouche et al.^11^ suggest using pre-dialysis BUN (blood urea nitrogen) < 35 mg/dL (urea < 75 mg/dL) at midweek as a target for dose adjustment. It is also worth recognizing that increasing the dialysis dose and maintaining BUN levels very close to physiological values are associated with greater fetal survival, gestational age, and fetal birth weight, and a reduced risk of polyhydramnios^10,11,18,83,84^.

Assymira et al.^84^ observed a significant correlation between pre-dialysis BUN and both fetal weight and gestational age. In the study by Luders et al.^18^, BUN appears as an independent variable for fetal outcome. It was also observed that patients with pre-dialysis BUN levels < 35 mg/dL measured midweek throughout pregnancy exhibited a 6.4-fold reduced risk of unfavorable fetal outcomes (fetal death and/or a fetal birth at less than 30 weeks of gestational age). The association between dialysis intensity and risk of preterm birth was analyzed by Oliverio et al.^85^, who were unable to observe a significant correlation between weekly dialysis dose or pre-dialysis BUN levels with the risk of preterm birth or cesarean delivery.

Nevertheless, for patients who started dialysis due to pregnancy, the administration of such an elevated dialysis dose did not prove beneficial. A comparative analysis of patients who started dialysis during pregnancy revealed that an American cohort underwent 15 ± 4 hours of dialysis per week *versus* 33 ± 6 hours per week in a Canadian cohort. Despite these differences in dialysis duration, both cohorts exhibited the same fetal outcomes, suggesting that patients who initiate dialysis during pregnancy do not require such high doses of dialysis. Additionally, the presence of residual clearance should guide the prescribed dialysis intensity. They also suggest that the pre-dialysis urea value from mondays, between 60–90 mg/dL, should be targeted in regimens of 6 weekly sessions^9^.

Polyhydramnios is relatively common in pregnant women on dialysis and is associated with an increased risk of preterm birth, premature rupture of membranes, and fetal death. It is explained by the increased fetal diuresis in the presence of higher levels of urea (osmotic diuresis). It has been demonstrated that increasing the dialysis dose, with the resulting reduction in maternal and fetal urea levels, usually reverses this complication^18^.

In PD, there is a scarcity of studies dedicated to the relationship between increasing the dialysis dose and obstetric outcomes, partly due to the less frequent occurrence of pregnancy in this modality (0.25% per year)^5,6^. Usually, particularly in the third trimester, PD prescription is optimized by using longer automated peritoneal dialysis sessions and a greater number of cycles with lower infusion volumes per cycle^86,87^. In the case of continuous ambulatory peritoneal dialysis, the infusion volume could be reduced with a higher number of exchanges. A recent meta-analysis has evidenced a higher risk of fetal growth restriction in this modality compared to HD. However, these findings are questionable, as the PD group consisted of case reports or case series^13^. In general, different authors suggest that patients on dialysis who become pregnant should remain on their dialysis method. Nevertheless, patients starting dialysis due to pregnancy are usually referred to HD^43^.

## Conclusion

Although fertility is reduced in women on dialysis, pregnancy remains a possibility, with birth rates increasing over time. Shared decision-making should be the goal for family planning of all women of childbearing age undergoing dialysis. Pregnancy on dialysis remains a challenging and high-risk clinical scenario, benefiting from multidisciplinary and interprofessional expertise to provide specialized care and increase birth rates, as observed in contemporary observational studies. Long-term studies on pregnancy outcomes in dialysis are still in progress, but the growing body of literature has been contributing to the formulation of recommendations on which we advise our patients. In particular, intensified dialysis is recommended for pregnant women on dialysis, as it is associated with better outcomes, including increased gestational age and birth weight.

## References

[1] Tangren J, Nadel M, Hladunewich M (2018). Pregnancy and end-stage renal disease. Blood Purif.

[2] Piccoli GB, Attini R, Vasario E, Conijn A, Biolcati M, D’Amico F (2010). Pregnancy and chronic kidney disease: a challenge in all CKD stages. Clin J Am Soc Nephrol.

[3] Wiles KS, Nelson-Piercy C, Bramham K (2018). Reproductive health and pregnancy in women with chronic kidney disease. Nat Rev Nephrol.

[4] Fayed A, Soliman A, Naguib M, Soliman M, Salaheldin M (2019). Ovarian reserve in an Egyptian cohort with end-stage kidney disease on hemodialysis and after successful kidney transplantation: a prospective study. Int Urol Nephrol.

[5] Shahir AK, Briggs N, Katsoulis J, Levidiotis V (2013). An observational outcomes study from 1966-2008, examining pregnancy and neonatal outcomes from dialysed women using data from the ANZDATA Registry. Nephrology (Carlton).

[6] Shah S, Christianson AL, Meganathan K, Leonard AC, Schauer DP, Thakar CV (2019). Racial Differences and Factors Associated with Pregnancy in ESKD Patients on Dialysis in the United States. J Am Soc Nephrol.

[7] Jesudason S, Grace BS, Mc Donald SP (2014). Pregnancy outcomes according to dialysis commencing before or after conception in women in ESRD. Clin J Am Soc Nephrol.

[8] Normand G, Xu X, Panaye M, Jolivot A, Lemoine S, Guebre-Egziabher F (2018). Pregnancy Outcomes in French Hemodialysis Patients. Am J Nephrol.

[9] Hladunewich M, Schatell D (2016). Intensive dialysis and pregnancy. Hemodial Int.

[10] Manisco G, Potì’ M, Maggiulli G, Di Tullio M, Losappio V, Vernaglione L (2015). Pregnancy in end-stage renal disease patients on dialysis: how to achieve a successful delivery. Clin Kidney J.

[11] Baouche H, Jais JP, Meriem S, Kareche M, Moranne O, Vigneau C (2023). Pregnancy in women on chronic dialysis in the last decade (2010–2020): a systematic review. Clin Kidney J.

[12] Yang LY, Thia EW, Tan LK (2010). Obstetric outcomes in women with end-stage renal disease on chronic dialysis: a review. Obstet Med.

[13] Piccoli GB, Minelli F, Versino E, Cabiddu G, Attini R, Vigotti FN (2016). Pregnancy in dialysis patients in the new millennium: a systematic review and meta-regression analysis correlating dialysis schedules and pregnancy outcomes. Nephrol Dial Transplant.

[14] Oliverio AL, Hladunewich MA (2020). End-stage kidney disease and dialysis in pregnancy. Adv Chronic Kidney Dis.

[15] Agrawal A, Wenger NK (2020). Hypertension during pregnancy. Curr Hypertens Rep.

[16] Braunthal S, Brateanu A (2019). Hypertension in pregnancy: pathophysiology and treatment. SAGE Open Med.

[17] Gojnic M, Todorovic J, Stanisavljevic D, Jotic A, Lukic L, Milicic T (2022). Maternal and fetal outcomes among pregnant women with diabetes. Int J Environ Res Public Health.

[18] Luders C, Titan SM, Kahhale S, Francisco RP, Zugaib M (2018). Risk factors for adverse fetal outcome in hemodialysis pregnant women. Kidney Int Rep.

[19] Battarbee AN, Sinkey RG, Harper LM, Oparil S, Tita ATN (2020). Chronic hypertension in pregnancy. Am J Obstet Gynecol.

[20] American College of Obstetricians and Gynecologists’ Committee on Practice Bulletins—Obstetrics. (2019). ACOG practice bulletin no. 203: chronic hypertension in pregnancy. Obstet Gynecol.

[21] Lecarpentier E, Tsatsaris V, Goffinet F, Cabrol D, Sibai B, Haddad B (2013). Risk factors of superimposed preeclampsia in women with essential chronic hypertension treated before pregnancy. PLoS One.

[22] Sibai BM (2002). Chronic hypertension in pregnancy. Obstet Gynecol.

[23] Panaitescu AM, Syngelaki A, Prodan N, Akolekar R, Nicolaides KH (2017). Chronic hypertension and adverse pregnancy outcome: a cohort study. Ultrasound Obstet Gynecol.

[24] Bateman BT, Heide-Jørgensen U, Einarsdóttir K, Engeland A, Furu K, Gissler M (2018). β-Blocker use in pregnancy and the risk for congenital malformations: an international cohort study. Ann Intern Med.

[25] Drambarean B, Mastalerz J, Wendt L, Toth-Manikowski S (2023). Pharmacotherapy considerations in pregnant patients on hemodialysis. Hemodial Int.

[26] Martinez A, Lakkimsetti M, Maharjan S, Aslam MA, Basnyat A, Kafley S (2023). Beta-blockers and their current role in maternal and neonatal health: a narrative review of the literature. Cureus.

[27] Abalos E, Duley L, Steyn DW (2014). Antihypertensive drug therapy for mild to moderate hypertension during pregnancy. Cochrane Database Syst Rev.

[28] Mito A, Murashima A, Wada Y, Miyasato-Isoda M, Kamiya CA, Waguri M (2019). Safety of amlodipine in early pregnancy. J Am Heart Assoc.

[29] Statement SMFM (2015). benefit of antihypertensive therapy for mild-to-moderate chronic hypertension during pregnancy remains uncertain. SMFM Publications Committee. Am J Obstet Gynecol.

[30] Magee LA, von Dadelszen P, Singer J, Lee T, Rey E, Ross S (2016). Do labetalol and methyldopa have different effects on pregnancy outcome? Analysis of data from the Control of Hypertension In Pregnancy Study (CHIPS) trial. BJOG.

[31] Sharma C, Soni A, Gupta A, Verma A, Verma S (2017). Hydralazine vs nifedipine for acute hypertensive emergency in pregnancy: a randomized controlled trial. Am J Obstet Gynecol.

[32] Magee LA, Cham C, Waterman EJ, Ohlsson A, von Dadelszen P (2003). Hydralazine for treatment of severe hypertension in pregnancy: meta-analysis. BMJ.

[33] Malhamé I, Dong S, Syeda A, Ashraf R, Zipursky J, Horn D (2023). The use of loop diuretics in the context of hypertensive disorders of pregnancy: a systematic review and meta-analysis. J Hypertens.

[34] Cooper WO, Hernandez-Diaz S, Arbogast PG, Dudley JA, Dyer S, Gideon PS (2006). Major congenital malformations after first-trimester exposure to ACE inhibitors. N Engl J Med.

[35] Gehlert J, Morton A (2021). Eplerenone as a treatment for resistant hypertension in pregnancy. Obstet Med.

[36] Sass N, Itamoto CH, Silva MP, Torloni MR, Atallah AN (2007). Does sodium nitroprusside kill babies? A systematic review. Sao Paulo Med J.

[37] Barrett PM, McCarthy FP, Evans M, Kublickas M, Perry IJ, Stenvinkel P (2020). Hypertensive disorders of pregnancy and the risk of chronic kidney disease: a Swedish registry-based cohort study. PLoS Med.

[38] Barton JR, O’brien JM, Bergauer NK, Jacques DL, Sibai BM (2001). Mild gestational hypertension remote from term: progression and outcome. Am J Obstet Gynecol.

[39] ACOG Practice Bulletin (2020). Gestational Hypertension and Preeclampsia: ACOG Practice Bulletin, Number 222. Obstet Gynecol.

[40] Peraçoli JC, Costa ML, Cavalli RC, Oliveira LG, Korkes HA, Ramos JGL (2023). Pré-eclampsia – Protocolo 2023.. Rede Brasileira de Estudos sobre Hipertensão na Gravidez;.

[41] Cornelis T, Spaanderman M, Beerenhout C, Perschel FH, Verlohren S, Schalkwijk CG (2013). Antiangiogenic factors and maternal hemodynamics during intensive hemodialysis in pregnancy. Hemodial Int.

[42] Costa ML, Cavalli RC, Korkes HA, Cunha EVD, Peraçoli JC (2022). Diagnosis and management of preeclampsia: suggested guidance on the use of biomarkers. Rev Bras Ginecol Obstet.

[43] Copur S, Berkkan M, Basile C, Cozzolino M, Kanbay M (2023). Dialysis in pregnancy: an update review. Blood Purif.

[44] Jong MFC, Hamersvelt HW, Empel WH, Nijkamp EJW, Lely AT (2022). Summary of the Dutch practice guideline on pregnancy wish and pregnancy in CKD. Kidney Int Rep.

[45] Fishel Bartal M, Sibai BM (2022). Eclampsia in the 21st century. Am J Obstet Gynecol.

[46] Mayo Clinic. Calcium and calcium supplements: achieving the right balance [Internet].[citado 2022Nov 1]..

[47] Vousden N, Lawley E, Seed PT, Gidiri MF, Goudar S, Sandall J (2019). Incidence of eclampsia and related complications across 10 low- and middle-resource geographical regions: secondary analysis of a cluster randomised controlled trial. PLoS Med.

[48] Edlow JA, Caplan LR, O’Brien K, Tibbles CD (2013). Diagnosis of acute neurological emergencies in pregnant and post-partum women. Lancet Neurol.

[49] Erez O, Romero R, Jung E, Chaemsaithong P, Bosco M, Suksai M (2022). Preeclampsia and eclampsia: the conceptual evolution of a syndrome. Am J Obstet Gynecol.

[50] Lisonkova S, Bone JN, Muraca GM, Razaz N, Wang LQ, Sabr Y (2021). Incidence and risk factors for severe preeclampsia, hemolysis, elevated liver enzymes, and low platelet count syndrome, and eclampsia at preterm and term gestation: a population-based study. Am J Obstet Gynecol.

[51] Guida JP, Surita FG, Parpinelli MA, Costa ML (2017). Preterm preeclampsia and timing of delivery: a systematic literature review. Rev Bras Ginecol Obstet.

[52] Magee LA, Brown MA, Hall DR, Gupte S, Hennessy A, Karumanchi AS (2022). The hypertensive disorders of pregnancy: the 2021 International Society for the Study of Hypertension in Pregnancy Classification, Diagnosis & Management Recommendations for International Practice. Pregnancy Hypertens.

[53] Van der Tuuk K, Holswilder-Olde Scholtenhuis MA, Koopmans CM, van den Akker ES, Pernet PJ, Ribbert LS (2015). Prediction of neonatal outcome in women with gestational hypertension or mild preeclampsia after 36 weeks of gestation. J Matern Fetal Neonatal Med.

[54] Nicolle LE, Gupta K, Bradley SF, Colgan R, DeMuri GP, Drekonja D (2019). Clinical practice guideline for the management of asymptomatic bacteriuria: 2019 update by the Infectious Diseases Society of America. Clin Infect Dis.

[55] Kwon YE, Oh DJ, Kim MJ, Choi HM (2020). Prevalence and clinical characteristics of asymptomatic pyuria in chronic kidney disease. Ann Lab Med.

[56] Cabiddu G, Castellino S, Gernone G, Santoro D, Moroni G, Giannattasio M (2016). A best practice position statement on pregnancy in chronic kidney disease. The Italian Study Group on Kidney and Pregnancy. J Nephrol.

[57] Smaill FM, Vazquez JC (2019). Antibiotics for asymptomatic bacteriuria in pregnancy. Cochrane Database Syst Rev.

[58] Minassian C, Thomas SL, Williams DJ, Campbell O, Smeeth L (2013). Acute maternal infection and risk of pre-eclampsia: a population-based case-control study. PLoS One.

[59] Kazemier BM, Koningstein FN, Schneeberger C, Ott A, Bossuyt PM, de Miranda E (2015). Maternal and neonatal consequences of treated and untreated asymptomatic bacteriuria in pregnancy: a prospective cohort study with an embedded randomised controlled trial. Lancet Infect Dis.

[60] Wing DA, Fassett MJ, Getahun D (2014). Acute pyelonephritis in pregnancy: a 18-year retrospective analysis. Am J Obstet Gynecol.

[61] Bookstaver PB, Bland CM, Griffin B, Stover KR, Eiland LS, McLaughlin M (2015). A review of antibiotic use in pregnancy. Pharmacotherapy.

[62] Boulay H, Mazaud-Guittot S, Supervielle J, Chemouny JM, Dardier V, Lacroix A (2021). Maternal, fetal and child consequences of immunosuppressive drugs during pregnancy in women with organ transplant: a review. Clin Kidney J.

[63] Attini R, Cabiddu G, Ciabatti F, Montersino B, Carosso AR, Gernone G (2023). Chronic kidney disease, female infertility, and medically assisted reproduction: a best practice position statement by the Kidney and Pregnancy Group of the Italian Society of Nephrology. J Nephrol.

[64] Haninger-Vacariu N, Herkner H, Lorenz M, Säemann M, Vychytil A, Jansen M (2020). Exclusion of pregnancy in dialysis patients: diagnostic performance of human chorionic gonadotropin. BMC Nephrol.

[65] Hladunewich MA (2017). Chronic kidney disease and pregnancy. Semin Nephrol.

[66] Zajdenverg L, Façanha C, Dualib P, Golbert A, Moisés E, Calderon I (2023). Rastreamento e diagnóstico da hiperglicemia na gestação. Diretriz Oficial da Sociedade Brasileira de Diabetes.

[67] Piccoli GB, Zakharova E, Attini R, Ibarra Hernandez M, Orozco Guillien A, Alrukhaimi M (2018). Pregnancy in chronic kidney disease: need for higher awareness. A pragmatic review focused on what could be improved in the different CKD stages and phases. J Clin Med.

[68] Esposito P, Garibotto G, Picciotto D, Costigliolo F, Viazzi F, Conti NE (2020). Nutritional challenges in pregnant women with renal diseases: relevance to fetal outcomes. Nutrients.

[69] Hanson MA, Bardsley A, De-Regil LM, Moore SE, Oken E, Poston L (2015). The International Federation of Gynecology and Obstetrics (FIGO) recommendations on adolescent, preconception and maternal nutrition: “Think Nutritional First. Int J Gynaecol Obstet.

[70] Reyes-López MA, Piccoli GB, Leone F, Orozco-Guillén A, Perichart-Perera O (2020). Nutrition care for chronic kidney disease during pregnancy: an updated review. Eur J Clin Nutr.

[71] Hofmeyr GJ, Lawrie TA, Atallah AN, Duley L, Torloni MR (2014). Calcium supplementation during pregnancy for preventing hypertensive disorders and related problems. Cochrane Database Syst Rev.

[72] Sekar A, Kaur T, Nalley JV, Rincon-Choles H, Jolly S, Nakhoul GN (2018). Phosphorus binders: the new and the old, and how to choose. Cleve Clin J Med.

[73] De-Regil LM, Palacios C, Ansary A, Kulier R, Peña-Rosas JP (2012). Vitamin D supplementation for women during pregnancy. Cochrane Database Syst Rev.

[74] KDIGO Clinical Practice Guideline for Anemia in Chronic Kidney Disease. (2012). Summary of Recommendation Statements. Kidney Int Suppl.

[75] Scholl TO, Reilly T (2000). Anemia, iron and pregnancy outcome. J Nutr.

[76] Coordenação-geral de gestão de protocolos clínicos e diretrizes e diretrizes terapêuticas – CGPCDT/DGITS/SECTICS/MS. (2023). Protocolo Clínico e Diretrizes Terapêuticas Anemia por Deficiência de Ferro.

[77] de Souza AI, Batista M, Ferreira LOC, Figueirôa JN (2004). Efetividade de três esquemas com sulfato ferroso para tratamento de anemia em gestantes. Rev Panam Salud Publica.

[78] Jose A, Mahey R, Sharma JB, Bhatla N, Saxena R, Kalaivani M (2019). Comparison of ferric carboxymaltose and iron sucrose complex for treatment of iron deficiency anemia in pregnancy – randomised controlled trial. BMC Pregnancy Childbirth.

[79] Chang JY, Jang H, Chung BH, Youn YA, Sung IK, Kim YS (2016). The successful clinical outcomes of pregnant women with advanced chronic kidney disease. Kidney Res Clin Pract.

[80] Barratt J, Dellanna F, Portoles J, Choukroun G, De Nicola L, Young J (2023). Safety of roxadustat versus erythropoiesis-stimulating agents in patients with anemia of non-dialysis-dependent or incident-to-dialysis chronic kidney disease: pooled analysis of four phase 3 studies. Adv Ther.

[81] Martimbianco ALC, Moreira RFC, Pacheco RL, Latorraca COC, Dos Santos APP, Logullo P (2023). Efficacy and safety of hemodialysis strategies for pregnant women with chronic kidney disease: systematic review. Semin Dial.

[82] Hladunewich MA, Hou S, Odutayo A, Cornelis T, Pierratos A, Goldstein M (2014). Intensive hemodialysis associates with improved pregnancy outcomes: a Canadian and United States cohort comparison. J Am Soc Nephrol.

[83] Luders C, Castro MC, Titan SM, De Castro I, Elias RM, Abensur H (2010). Obstetric outcome in pregnant women on long-term dialysis: a case series. Am J Kidney Dis.

[84] Asamiya Y, Otsubo S, Matsuda Y, Kimata N, Kikuchi KAN, Miwa N (2009). The importance of low blood urea nitrogen levels in pregnant patients undergoing hemodialysis to optimize birth weight and gestational age. Kidney Int.

[85] Oliveiro AL, Bragg-Gresham JL, Admon LK, Nunes JAW, Saran R, Heung M (2020). Obstetric deliveries in US women with ESKD: 2002-2015. Am J Kidney Dis.

[86] Seong Lim CT, Wah FK (2018). Pregnancy and peritoneal dialysis: an updated review. EMJ Nephrol.

[87] Batarse RR, Steiger RM, Guest S (2015). Peritoneal dialysis prescription during the third trimester of pregnancy. Perit Dial Int.

