# Supplementary material for: Pregnancy in patients with chronic kidney disease undergoing dialysis
Source: J Bras Nefrol. 2024 Nov 8;46(4):e20240067. doi: 10.1590/2175-8239-JBN-2024-0067en (PMC11548865; doi:10.1590/2175-8239-JBN-2024-0067en)
Supplement: Translation

# Translation: Gestação de paciente com doença renal crônica em diálise

**DOI:** 10.1590/2175-8239-JBN-2024-0067pt

**Authors:** Fernanda Salomão Gorayeb-Polacchini, Ana Flavia Moura, Claudio Luders, José Andrade Moura, Juliana El Ghoz Leme, Dirceu Reis da Silva

**Affiliations:** 1 Sociedade Brasileira de Nefrologia, São Paulo, SP, Brasil.; 2 Hospital de Base de São José do Rio Preto, São José do Rio Preto, SP, Brasil.; 3 Escola Bahiana de Medicina e Saúde Pública, Salvador, BA, Brasil.; 4 Hospital Sírio-Libanês, São Paulo, SP, Brasil.; 5 Nefroclínicas Curitiba, Curitiba, PR, Brasil.; 6 Hospital de Clínicas de Porto Alegre, Porto Alegre, RS, Brasil.

**Keywords:** Doença Renal Crônica, Diálise, Gestação, Complicações da Gravidez, Pré-Eclâmpsia

## Abstract

As mulheres portadoras de doença renal crônica apresentam menor probabilidade de engravidar e são mais propensas a complicações gestacionais quando comparadas a pacientes com função renal normal, sendo, portanto, consideradas gestantes de alto risco materno e fetal. Ao longo dos anos, verificou-se aumento da incidência de gestações em pacientes em diálise e melhora do desfecho materno e fetal. Acredita-se que a otimização do atendimento obstétrico e neonatal, o ajuste do tratamento dialítico (em especial, o aumento do número de horas e da frequência semanal das sessões de diálise) e o uso de agentes estimuladores da eritropoiese têm proporcionado melhor controle metabólico, volêmico, pressórico, eletrolítico e da anemia. Este artigo de revisão tem o objetivo de analisar os desfechos gestacionais em pacientes com doença renal crônica em diálise e revisar a condução médica nefrológica nesse cenário. Pelo crescente interesse pelo tema, as recomendações clínicas para a prática assistencial têm ganhado em consistência tanto no manejo medicamentoso quanto no manuseio dialítico, aspectos que tratamos na presente revisão.

## Introdução

A doença renal crônica (DRC) atinge cerca de 3% das mulheres em idade reprodutiva e é um fator para alto risco gestacional. Com a diminuição da taxa de filtração glomerular, observa-se progressivamente a diminuição da fertilidade e o aumento do risco de desfechos desfavoráveis da gestação, com poucos estudos explorando os mecanismos fisiopatológicos subjacentes a essa associação^1,2^. A hiperprolactinemia e os níveis séricos baixos de estradiol observados em pacientes em diálise estão ligados à alteração do hormônio liberador de gonadotrofina e do eixo hipotálamo-hipófise-gonadal, e podem justificar dificuldade de ovulação. Há ainda resultados conflitantes associando os baixos níveis séricos de hormônio anti-Mulleriano observados em pacientes em diálise com o prejuízo da fertilidade^3,4^.

## Discussão

### Epidemiologia

Ao longo dos anos, tem crescido o interesse pela fertilidade e pela gestação em pacientes em diálise, viabilizando a progressiva melhora do prognóstico materno e fetal. Na Tabela 1, está apresentada a evolução do aumento da incidência da taxa de gravidez nessa população^5,6^. Uma coorte americana demonstrou que mulheres caucasianas em diálise têm maior incidência de gravidez, sendo que o diabetes *mellitus* (DM), a modalidade de diálise peritoneal (DP) e o maior tempo em diálise são características adicionais que reduzem a fertilidade^6^. Gestações iniciadas em pacientes com doença renal crônica ainda sem tratamento dialítico têm maior taxa de fetos nascidos vivos (91%) comparadas àquelas que as iniciam durante a fase dialítica (66%)^7^.

**Tabela 1:** Incidência das gestações em diálise ao longo dos anos

Estudo	Ano	Incidência
ANZDATA^5^	1976–1985	0,54 por 1.000 pacientes-ano
	1986–1995	0,67 por 1.000 pacientes-ano
	1996–2008	3,3 por 1.000 pacientes-ano
Shah et al.^6^	2005–2013	17,8 por 1.000 pacientes-ano
		40,9 por 1.000 pacientes-ano (pacientes de 20–24 anos)

Vários aspectos parecem explicar o aumento da incidência e dos melhores desfechos das gestações ao longo dos anos: 1) melhor manejo da anemia com agentes estimuladores da eritropoiese (AEE), 2) melhora do tratamento hemodialítico com membranas biocompatíveis, 3) aumento de dose semanal de diálise, por maior frequência ou tempo de sessão, proporcionando menor exposição ao ambiente urêmico e melhor controle volêmico e pressórico, e 4) avanços da monitorização e do tratamento obstétricos e neonatais^1,2,5,8,9,10^.

### Desfechos e Complicações Relacionadas à Gestação

A partir de revisão sistemática de 14 estudos entre os anos de 2010–2020, com 11 países participantes (de 6 continentes), e incluindo 2.754 gestações em 2.364 pacientes em programa de diálise, obteve-se dados abrangentes do cenário da gestação em diálise da última década (Tabela 2)^11^. A principal modalidade de terapia renal substitutiva foi a hemodiálise (HD), em 92,6% dos casos^11^. Diferentemente de revisões mais antigas^5,6,12,13^, este estudo reuniu maior número de gestações e evidenciou melhor prognóstico^11^.

**Tabela 2:** Desfechos e complicações da gestação em mulheres em diálise^11^

Desfechos e complicações	Resultados
**Desfechos relacionados à gestação**	
Idade gestacional ao nascer	25 a 36 semanas
Parto cesárea	9% a 100%
Aborto espontâneo	16,9%
Natimorto	8,3%
Polidrâmnio	17,7%
Interrupção da gestação	5,2%
**Causas de interrupção da gestação**	
Desordens hipertensivas da gestação	17,1%
Trabalho de parto prematuro	8,1%
Crescimento intrauterino retardado	6,5%
Descolamento prematuro de placenta	3,2%
Insuficiência cervical	2,3%
Sofrimento fetal	2,3%
Cesariana prévia	2,3%
**Complicações maternas**	
Hipertensão arterial	7,7%
Pré-eclâmpsia	11,9%
Eclâmpsia	0,7%
Síndrome HELLP	0,7%
Anemia	3,9%
Diabetes gestacional	0,5%
Peritonite	0,2%
Colestase	0,7%
Morte materna*	0%

HELLP: hemólise, elevação de enzimas hepáticas, e plaquetopenia.

*Da gestação até 42 dias após o parto (OMS).

Nessa população, uma complicação a ser acompanhada é o polidrâmnio, que pode atingir até 53% das gestantes em alguns estudos, podendo constituir indicação de aumento da dose e tempo de diálise, bem como ajuste do peso seco^14^.

## Complicações Maternas Durante a Gestação

Na Tabela 2, encontram-se também listadas as principais complicações maternas em gestantes em tratamento dialítico^11^, destacando-se as síndromes hipertensivas, que ao longo dos últimos anos tiveram redução na incidência das suas formas graves em decorrência do emprego de diálise mais intensiva e melhor acompanhamento obstétrico^11,12,15,16^.

Assim como em pacientes que não estão em tratamento dialítico, há maior risco de complicações maternas e fetais em pacientes em diálise primigestas, com maior idade, obesas e com diagnóstico prévio de hipertensão arterial (HA), lúpus eritematoso sistêmico e DM^17,18^.

## Síndromes Hipertensivas da Gestação

As síndromes hipertensivas são as causas mais comuns de óbito entre gestantes por elevarem o risco cardiovascular (CV) materno, inclusive após a gestação^15,16,19^. As recomendações para o manejo dessas condições para gestantes em diálise são em grande parte derivadas daquelas para gestantes que não estão em tratamento dialítico, ajustadas às particularidades da DRC.

### Hipertensão Arterial Crônica (HAC)

A hipertensão arterial crônica (HAC) é definida pela elevação da pressão arterial sistólica (PAS) ≥ 140 mmHg ou da pressão arterial diastólica (PAD) ≥ 90 mmHg, em pelo menos duas medidas realizadas com um intervalo mínimo de 4 horas, antes da 20ª semana de gestação, ou que persista por 12 semanas ou mais após o parto. Em pacientes que apresentam elevação severa da pressão arterial (PAS ≥ 160 mmHg ou PAD ≥ 110 mmHg), permite-se fazer o diagnóstico de HAC com apenas uma medida, a fim de facilitar o início da terapia^20,21^. Até o momento, não há consenso acerca da meta pressórica ideal para gestantes hipertensas. Diferentes alvos são propostos, parecendo razoável buscar uma PAS < 140 mmHg e PAD < 90 mmHg para gestantes hipertensas em diálise.

O risco CV e a mortalidade em gestantes hipertensas são maiores quando comparados a gestantes normotensas, e o controle pressórico reduz tal risco^22^. Além disso, observa-se uma prevalência aumentada de diabetes gestacional em mulheres hipertensas em relação àquelas normotensas, o que parece ser justificado pelos fatores de risco em comum^23^.

Para reduzir riscos obstétricos, mulheres hipertensas precisam, ainda na fase de pré-concepção, de avaliação e adoção de medidas para o controle pressórico. Condições como obesidade e DM, bem como adequações no estilo de vida, devem ser abordadas e controladas antes da gravidez. Além disso, o esquema anti-hipertensivo deve ser revisado, com atenção para as medicações contraindicadas ou com pouca evidência de segurança na gravidez^20^. Os anti-hipertensivos disponíveis no Brasil que são recomendados para uso em gestantes são: metoprolol ou pindolol, nifedipino de longa duração, metildopa e hidralazina.

Os betabloqueadores são os anti-hipertensivos mais utilizados na gestação. Embora alguns estudos tenham sugerido associação dessa classe com restrição do fluxo placentário, diminuição do crescimento intrauterino e do peso fetal, malformações fetais, hipoglicemia neonatal e aumento da mortalidade perinatal (como, por exemplo, a verificada com atenolol), a maioria dos estudos não demonstrou excesso do risco de desfechos obstétricos desfavoráveis em comparação com outros anti-hipertensivos liberados para gestantes^24,25^. Recomenda-se em especial a utilização de labetalol (não disponível no Brasil), pindolol ou metoprolol. O propranolol tem resultados conflitantes entre os estudos quanto a complicações neonatais, porém com possibilidade de uso na lactação^26^.

Os bloqueadores dos canais de cálcio também são amplamente utilizados em gestantes hipertensas, principalmente na segunda metade da gestação, por seus efeitos tocolíticos. Entre eles, o nifedipino (apresentações de liberação lenta ou ação intermediária) é o mais utilizado^27^. Apesar de amplamente utilizado na população hipertensa em geral, o anlodipino é relativamente pouco empregado nas gestantes devido à escassez de dados na literatura, ainda que exista evidência de segurança em estudo de 48 pacientes^28^. Os não diidropiridínicos também são eficazes na gestação, porém exigem cautela no uso por estarem associados a interferência na frequência cardíaca fetal^29^.

A metildopa é o anti-hipertensivo utilizado há mais tempo na gravidez, com melhor conhecimento sobre seus potenciais efeitos adversos. Demonstrou-se menor risco da metildopa em relação aos demais anti-hipertensivos liberados na gestação, inclusive quando comparada ao labetalol^30^. As desvantagens da metildopa é seu tempo retardado de início de ação (3–6 horas) e sua baixa potência, requerendo muitas vezes doses elevadas (aumento do risco de hepatotoxicidade, anemia hemolítica e efeitos sedativos). Além disso, sua moderada dialisância e o curto intervalo posológico acarretam limitações em seu uso como droga ideal na gestação em diálise. No conjunto, é preferível utilizar a metildopa como terapia combinada^20,25^.

A hidralazina pode ser utilizada na gestação tanto em sua apresentação oral quanto endovenosa. Sua principal indicação é no tratamento agudo de picos hipertensivos, pelo seu rápido início de ação (10–20 minutos)^31^. Nesses casos, a incidência de efeitos adversos é maior, chegando a afetar cerca de 50% das pacientes^20^, devendo ser dada especial atenção à hipotensão, aumentando o risco materno e fetal determinado pela redução do fluxo uteroplacentário. Recomenda-se então a infusão intravenosa intermitente de 5–10 mg de hidralazina a cada 20–40 minutos, não ultrapassando a dose máxima de 20 mg^32^. Nos esquemas de manutenção, a hidralazina é mais utilizada em terapias combinadas, principalmente com betabloqueadores, que reduzem alguns de seus efeitos adversos.

Os diuréticos tiazídicos ou de alça são seguros na gestação, em que pese a preocupação com seus efeitos na volemia materna e risco de redução da perfusão uteroplacentária, em particular nas duas primeiras semanas de uso, quando ocorre maior redução do volume plasmático^20,33^. Em gestantes portadoras de DRC, seu uso costuma ser seguro, considerando a participação da hipervolemia na HA. No contexto da gestação em mulheres em tratamento dialítico, o emprego de diálise intensiva reduz a necessidade de emprego de diuréticos, que costuma ser reservado a gestantes com insuficiência cardíaca ou DRC não dialítica. Em casos refratários de hipervolemia, pode ser utilizada a combinação de diuréticos^33^.

Os inibidores do sistema renina-angiotensina-aldosterona (iSRAA) apresentam elevada associação com anormalidades renais no feto quando utilizados na segunda metade da gestação^34^. Apesar de seus riscos no primeiro trimestre ainda não estarem bem definidos, a recomendação é de que iSRAA devem ser substituídos ainda no período pré-concepcional ou assim que diagnosticada a gravidez^34^.

Como regra, os antagonistas do receptor mineralocorticoide não são recomendados durante a gestação, principalmente devido à escassez de dados na literatura e à preocupação com os efeitos antiandrogênicos sobre o desenvolvimento fetal. Novas drogas dessa classe têm menor efeito antiandrogênico, mas com pouca experiência de uso em gestantes^35^.

O nitroprussiato de sódio pode ser utilizado em situações específicas, como emergências hipertensivas no pré-parto, sempre por curto período, pesando o risco-benefício, devido a evidências inconclusivas sobre o risco na gestação^36^.

### Hipertensão Gestacional

A hipertensão gestacional consiste na ocorrência de HA iniciada com 20 semanas ou mais de gestação e que não apresentem sinais ou sintomas sugestivos de pré-eclâmpsia (PE)^15^. Caso a paciente permaneça com elevação da PA por 12 semanas ou mais após o parto, o diagnóstico de hipertensão gestacional deve ser substituído pelo de HAC.

Em geral, classifica-se a hipertensão gestacional em severa (PAS ≥ 160 mmHg ou PAD ≥ 110 mmHg), e não severa (PAS < 160 mmHg ou PAD < 110 mmHg), sendo que a severa está associada a maior morbidade materna e fetal e a maior risco de desenvolvimento materno de HAC^37^.

Entre as gestantes com hipertensão gestacional, 10–50% delas desenvolverá PE entre 1–5 semanas após o diagnóstico^38^, não havendo certeza sobre ser ou não um estágio inicial da PE. A hipertensão gestacional severa e a PE devem ser tratadas de forma semelhante, com início ou ajuste imediato do esquema anti-hipertensivo e acompanhamento intra-hospitalar^39^.

### Pré-Eclâmpsia

A PE corresponde ao quadro de HA iniciado após a 20ª semana de gestação, associada à proteinúria (≥ 300 mg/24 horas ou relação proteína/creatinina urinária ≥ 0,3 mg/mg) ou disfunção de órgãos-alvo como plaquetopenia (< 150.000/mm^3^), disfunção hepática (transaminases TGO ou TGP > 40 UI/L), insuficiência renal (creatinina > 1 mg/dL), ou edema pulmonar. Além disso, sinais de disfunção placentária (como restrição de crescimento fetal e/ou alterações dopplervelocimétricas fetais) também devem chamar atenção para o diagnóstico de PE, mesmo na ausência de proteinúria. Vale ressaltar que a PE pode surgir durante a gestação ou mesmo após o parto, embora seja rara a partir do segundo dia pós-parto^40^.

O diagnóstico da PE é particularmente desafiador em gestantes em diálise dada a alta prevalência de HAC e aumento prévio da creatinina e proteinúria, sendo necessário monitorar o desenvolvimento das manifestações extrarrenais (alterações hematológicas, hepáticas e neurológicas), além das manifestações fetais associadas^41^. Há carência de recursos diagnósticos mais orientadores, entre os quais cita-se o uso de biomarcadores como fatores angiogênicos (por exemplo a relação sFlt-1/PlGF), que pode auxiliar na condução de casos duvidosos, principalmente a fim de excluir o diagnóstico em casos leves e moderados, poupando assim algumas gestantes de intervenções desnecessárias^42^.

A PE pode ser classificada de acordo com a idade gestacional no diagnóstico, sendo precoce (<34 semanas, associada a pior prognóstico) ou tardia (≥34 semanas)^41^.

Em mulheres com HAC prévia, a PE sobreposta é caracterizada por: 1) aumento dos níveis pressóricos, exigindo ajuste do esquema anti-hipertensivo, 2) aumento da proteinúria já detectada na primeira metade da gravidez, 3) ocorrência de disfunção de órgãos-alvo, ou 4) presença de sinais de disfunção placentária, como restrição de crescimento fetal e/ou alterações dopplervelocimétricas fetais^21,41^.

Antes denominada PE grave, a “PE com sinais de gravidade” (ou deterioração clínica e/ou laboratorial) é definida pela presença de: 1) crise hipertensiva com PA ≥ 160 e/ou 110 mmHg, persistente após 15 minutos; 2) emergência hipertensiva; 3) iminência de eclâmpsia; 4) eclâmpsia; 5) síndrome HELLP (hemólise, elevação de enzimas hepáticas, e plaquetopenia); 6) injúria renal aguda com creatinina ≥ 1,2 mg/dL ou oligúria < 500 mL/24h; 7) dor torácica; ou 8) edema pulmonar^40^.

A PE na gestante em diálise aumenta o risco de resultado fetal desfavorável em mais de 20 vezes, sendo o principal determinante da evolução^18^. Assim, estratégias de prevenção e diagnóstico, e protocolos específicos de seguimento, conduzidos por equipes multiprofissionais experientes, são fundamentais para um desfecho fetal adequado^43,44^.

O benefício do uso de ácido acetilsalicílico para a prevenção de PE em gestantes é bem demonstrado, motivo pelo qual deve-se empregá-lo nas gestantes em diálise, em que pese a inexistência de estudos nessa população, satisfeito que não exista contraindicação específica ao uso. As doses recomendadas variam entre 75–162 mg ao dia (sendo 100 mg a mais empregada do Brasil). Recomenda-se o início dessa profilaxia entre a 12ª e 16ª semanas de gestação, ou até a 28ª semana em casos de diagnóstico tardio^45^. O emprego de baixas doses de ácido acetilsalicílico não se associou à maior taxa de complicações no parto, sugerindo-se que possa ser mantido até o parto ou interrompido de 5–10 dias antes^45^.

Outra medida recomendada é a suplementação de cálcio, em especial nas populações com baixa ingesta (como a brasileira), iniciando no primeiro trimestre e mantendo-a até o fim da gestação, na dose recomendada de carbonato de cálcio 1-2 g/dia, ou citrato de cálcio 2–4 g/dia, fracionada em 3 tomadas^40^. Há melhor absorção quando empregado em pequenas doses (500 mg), principalmente nas refeições (afora o citrato de cálcio, que pode ser tomado longe das refeições, e é a forma recomendada para pacientes com distúrbios de absorção)^46^.

Todas as gestantes com PE com sinais de gravidade ou com hipertensão de difícil controle, ainda que assintomáticas, têm indicação de usar sulfato de magnésio antes do parto, de forma a prevenir crise convulsiva materna (eclâmpsia)^40^. Considerando que o magnésio é excretado por via renal, há risco de toxicidade potencialmente fatal (depressão respiratória ou do sistema nervoso central, e arritmias) em gestantes em diálise. Sendo assim, nesses casos deve-se administrar metade da dose preconizada e reduzir a velocidade de infusão, com monitorização criteriosa dos níveis séricos de magnésio e de sinais clínicos de toxicidade (depressão respiratória e reflexo tendinoso abolido). Frente a suspeita de intoxicação, avalia-se a necessidade da administração de gluconato de cálcio intravenoso para antagonizar os efeitos deletérios do magnésio^45^.

Por definição, as alterações associadas à PE evoluem para a resolução após o parto.

### Eclâmpsia

A eclâmpsia é definida pelo quadro não explicado de crise convulsiva ou coma em gestantes portadoras de síndrome hipertensiva da gestação, podendo existir sinais e sintomas precursores da crise convulsiva (como cefaleia intensa, alterações visuais, dor em quadrante superior do abdome, e pico hipertensivo)^45^. De prevalência decrescente em países desenvolvidos, mantém-se comum em alguns países em desenvolvimento. A eclâmpsia é a principal complicação da pré-eclâmpsia, e ocorre em 2-3% das gestantes que apresentam PE com sinais de gravidade, em especial em gestantes com menos de 20 anos ou mais de 35 anos^47^. Ainda que 60% dos casos de eclâmpsia ocorram durante a gestação, há aqueles que se desenvolvem no intraparto ou no pós-parto (90% destes na primeira semana pós-parto)^44^.

Crise tônico-clônica generalizada, de início súbito e sem causas identificadas, é a apresentação mais típica, sendo menos comuns crises focais, multifocais ou até mesmo o estado de coma^45^. Nas gestantes com crise tônico-clônica generalizada que evoluem sem déficit neurológico não é necessária avaliação diagnóstica adicional, que deve ser reservada para os casos atípicos (gestantes sem síndromes hipertensivas diagnosticadas ou com déficits neurológicos)^48^.

O sulfato de magnésio é a droga de escolha para a prevenção e tratamento da eclâmpsia, sendo também indicado na presença de pródromos^40^. Nas pacientes assintomáticas, não existe consenso quanto ao uso profilático de sulfato de magnésio, ainda que se postule profilaxia em todas as gestantes com PE, considerando a baixa toxicidade na população geral^49^. Sendo assim, seu uso também deve ser considerado em gestantes em diálise, respeitados os cuidados pelo maior risco de intoxicação por magnésio^45^.

O tratamento da crise convulsiva deve associar-se ao controle da HA e preservar o binômio materno-fetal, adotando-se medidas como a garantia da oxigenação materna (medida da oximetria, uso de oxigênio suplementar), correção da acidose, e minimização do risco de broncoaspiração (posicionar a gestante em decúbito lateral, aspirar secreções e inserir protetor bucal) durante a convulsão. Frente a PA ≥ 160/110 mmHg, deve-se utilizar preferencialmente hidralazina endovenosa ou nifedipino oral; o nitroprussiato de sódio pode ser utilizado em especial para edema pulmonar associado a cardiopatia, por curto período (6–12h), considerando o risco pouco documentado de toxicidade neurológica fetal^31,40,49^.

### Síndrome HELLP

Em geral, a síndrome HELLP ocorre entre as 28ª e 36ª semanas de gestação e o sintoma clínico mais comum é dor abdominal em quadrante superior, principalmente em epigástrio. Consiste na presença de 1) hemólise, reconhecida por desidrogenase láctica (DHL) duas vezes ou mais o limite superior da normalidade, bilirrubina indireta sérica ≥1,2 mg/dL, haptoglobina <25 mg/dL ou presença de esquizócitos em esfregaço periférico; 2) comprometimento hepático, traduzido por transaminases (TGO e/ou TGP) > 70 UI/L; e 3) plaquetopenia (<100.000 mm^3^)^40^.

Associada a elevado risco de complicações obstétricas, a síndrome HELLP deve receber manejo em ambiente hospitalar, com o tratamento imediato da HA severa. O sangramento hepático é comum, devendo ser considerado em pacientes com dor abdominal intensa no quadrante superior, e se confirmado, deve-se realizar a interrupção da gestação via cesariana assim que obtida a estabilidade hemodinâmica. Para prevenir convulsões maternas e oferecer neuroproteção fetal, o sulfato de magnésio deve ser iniciado de modo precoce e mantido por 24–48 horas após o parto^50^.

### Conduta Obstétrica na Pré-Eclâmpsia

Em mulheres com PE com idade gestacional inferior a 23 semanas, a conduta expectante associa-se a alta mortalidade perinatal e morbimortalidade materna. Portanto, diante de quadros de PE com sinais de gravidade (deterioração clínica e/ou laboratorial), recomenda-se a interrupção da gestação, uma vez que a viabilidade neonatal é baixa. Evidentemente, tal decisão deve ser compartilhada com a família^51^.

Na PE com idade gestacional entre 23–34 semanas, considerando o ônus de prematuridade, sugere-se atendimento em centro especializado, buscando-se preservar o feto. Na PE com sinais de gravidade, após a estabilização materna^39^, recomenda-se a interrupção da gestação ao se atingir 34 semanas, considerando a maior morbidade materna e fetal após essa idade gestacional^39^. Na ausência de sinais de gravidade da PE, sendo a idade gestacional entre 34–37 semanas, admite-se postergar o encerramento da gestação até a 37ª semana, em centro especializado^40,52^.

Na impossibilidade de garantir o bem-estar materno-fetal, deve-se indicar a interrupção da gestação, independentemente da idade gestacional^45^. Os critérios para interrupção são: 1) síndrome HELLP; 2) iminência de eclâmpsia refratária ao tratamento; 3) eclâmpsia; 4) descolamento prematuro de placenta; 5) HA refratária ao tratamento com três drogas anti-hipertensivas; 6) edema pulmonar/comprometimento cardíaco; 7) alterações laboratoriais progressivas; 8) elevação progressiva de ureia e creatinina, oligúria e anasarca; 9) hematoma ou rotura hepática; e 10) alterações na vitalidade fetal^53^.

## Infecção do Trato Urinário na Gestação

Comum na gestação, a infecção do trato urinário (ITU) pode apresentar-se como cistite ou pielonefrite, sendo esta mais comum em gestantes, devido ao relaxamento da musculatura lisa com subsequente dilatação ureteral e ao efeito compressivo do útero gravídico sobre a bexiga e o ureter^54^. Quando presente diurese residual nas gestantes em diálise, a apresentação clínica é similar às que não estão em tratamento dialítico, ao passo que pode ocorrer apenas dor abdominal e febre naquelas anúricas^55^.

Mais frequente no primeiro trimestre da gestação, a bacteriúria assintomática ocorre em 2–7% das gestantes^54^, e pode evoluir se não tratada para quadros sintomáticos em até 35% das mulheres, sendo de especial risco aquelas com antecedentes de ITU, aborto prévio^56^, portadoras de DM ou de doença renal policística autossômica dominante, e naquelas de baixo nível socioeconômico^54,57^. Recomenda-se o rastreio com urocultura na primeira consulta de pré-natal ou no primeiro trimestre, sendo controversa a periodicidade de novas avaliações; posicionamento italiano recomenda coleta mensal em pacientes de alto risco^56^. O tratamento parece estar associado a menor risco de pielonefrite, prematuridade, baixo peso ao nascer, mortalidade perinatal^57^ e PE^58^, ainda que nem todos os estudos demonstrem a mesma associação^54,59^.

Presente em até 2% das gestações, a pielonefrite é mais frequente no segundo e terceiro trimestres, podendo estar associada à morbidade materna e à prematuridade^60^. Considerando o risco materno e fetal, e a maior associação com anormalidades estruturais do trato urinário^54^, recomenda-se que gestantes em diálise com pielonefrite sejam atendidas em hospital, com antibiótico endovenoso, e submetidas a ultrassonografia do aparelho urinário quando há antecedentes de pielonefrite prévia, litíase renal, imunossupressão, e doença urológica^56^.

O manejo de cistite em gestantes em diálise é feito pelo emprego de antibióticos, preferentemente beta-lactâmicos, cefalosporinas de primeira ou segunda geração, ou fosfomicina, sendo reservado o emprego de cefalosporinas de terceira geração, piperacilina-tazobactam ou carbapenêmicos para casos de pielonefrite. Os antibióticos aminoglicosídeos, fluoroquinolonas, nitrofurantoína, sulfametoxazol-trimetoprim e tetraciclina devem ser evitados na gestante em diálise, pela associação com teratogenicidade ou toxicidade fetal^56,61^.

## Desfechos e Complicações Neonatais

As maiores complicações neonatais em gestações de pacientes em diálise incluem aborto, óbito fetal, prematuridade e baixo peso ao nascer^11^.

Tem-se assistido ao aumento da taxa de nascidos vivos em razão da maior oferta de horas e dias de diálise, bem como da melhoria dos tratamentos pré-natal e neonatal^7,11^. Existe ainda grande diferença entre países, e mesmo entre serviços de diálise, em relação à taxa de nascidos vivos e prematuridade^11^, presumivelmente em função da diversidade de práticas de diálise^11,13^, das diferenças populacionais e das desigualdades de atendimento obstétrico^9^. Acrescente-se que a taxa de nascidos vivos é maior em gestações de mulheres com DRC pré-dialítica, e mesmo naquelas em diálise com maior função renal residual^7^. Na Tabela 3 estão listadas as frequências das complicações neonatais principais^11^.

**Tabela 3:** Desfechos e complicações neonatais em gestações de pacientes em diálise^11^

Complicações e desfechos neonatais	Resultados
Nascidos vivos	71,4%
Peso ao nascer	590 a 3500 g
Prematuridade	82,8%
Necessidade de UTI neonatal	65,5%
Pequeno para idade gestacional	32%
SARA	2,4%
Mortes neonatais*	7,6%
Mortes perinatais^#^	15,3%

UTI: unidade de terapia intensiva, SARA: síndrome da angústia respiratória aguda.

*Mortes neonatais: óbitos entre o 1^o^ e 28^o^ dia após o nascimento.

#Mortes perinatais: óbitos entre a 22ª semana de gestação e o 6^o^ dia completo de vida.

Em estudo de gestações de pacientes em diálise, reconheceu-se 0,3% de malformações fetais, o que sugere que as malformações fetais não são mais frequentes em gestantes em diálise. Achados incluem: atresia duodenal, fenda palatina, anormalidades na orelha, hérnia abdominal, retardos mentais, retinopatia, glaucoma, dilatação pielocalicial, entre outras^11^.

## Tratamento não Dialítico Durante a Gestação

### Orientações pré-concepção

Toda paciente em tratamento dialítico em idade fértil deve ser informada sobre as perspectivas de uma gestação nesse contexto, incluindo prognóstico materno-fetal, assim como deve ser orientada sobre estratégias de contracepção. O adequado controle de doenças crônicas, como HA, DM e doenças autoimunes, é essencial para favorecer um melhor desfecho.

Em mulheres em idade fértil, com desejo de gestar, sem idade materna avançada e sem contraindicação a transplante, recomenda-se aguardar a realização de transplante renal, em razão dos melhores desfechos da gestação com enxerto funcionante^62^. A fertilidade é restabelecida pelo menos parcialmente após o transplante renal, sendo a melhor chance de uma gestação com sucesso associada à menor idade materna, normotensão, na ausência de obesidade ou proteinúria, com pelo menos dois anos transcorridos após o transplante, com função renal normal e sem nenhum episódio prévio de rejeição. Esse perfil ideal pode não ser plenamente exigido em pacientes que precisem esperar por um enxerto de doador falecido, pois o tempo de espera em lista pode impactar as decisões acerca do planejamento concepcional. Ainda assim, mesmo em condições ideais, o risco de complicações ainda é maior que o da população em geral^62^.

As pacientes em diálise ou transplantadas renais podem optar pela reprodução assistida, reconhecido, entretanto, o maior risco de sangramento, trombose e infecção em caso de gravidez gemelar. Ressalte-se que a hiperestimulação ovariana pode levar à piora da função renal em pacientes nefropatas crônicas e aumentar o risco de síndromes hipertensivas da gestação^63^.

### Tratamento Pós-Concepção

Para o diagnóstico de gravidez nas mulheres em diálise, há limitação do emprego da dosagem do *beta*-HCG, que costuma estar aumentado em pacientes com DRC avançada, proporcionando um eventual resultado falso positivo, e por isso é necessário complementar a avaliação com o exame de ultrassonografia obstétrica^64^.

Na ocorrência de uma gestação: 1) deve-se acionar a equipe multidisciplinar do serviço de diálise; 2) orientar o aumento do tempo e da frequência das sessões de diálise; 3) encaminhar para o pré-natal de alto risco; 4) avaliar as medicações em uso, com suspensão das medicações com potencial teratogênico; 5) avaliar a carteira vacinal; e 6) estabelecer um plano individual de monitorização clínica e laboratorial^43^.

O seguimento obstétrico também inclui vigilância constante do crescimento e desenvolvimento fetal e rastreio para malformações, assim como das condições placentárias. A via de parto deve ser definida pela indicação obstétrica, não sendo contraindicado o parto vaginal para as mulheres com doença renal^65^.

Recomenda-se que a periodicidade das consultas de pré-natal de alto risco seja de (pelo menos) uma vez ao mês durante o primeiro trimestre, a cada duas semanas durante o segundo trimestre e semanalmente durante o terceiro trimestre, período em que ocorrem maiores mudanças no *status* volêmico e maior risco de complicações relacionadas à PA^65^.

Os exames laboratoriais devem ser monitorados regularmente (pelo menos) a cada duas semanas, especialmente os níveis de ureia, bicarbonato, eletrólitos, glicose, hemograma, cálcio e fósforo. Após a 20^a^ semana, devem ser incluídas provas hepáticas e marcadores de hemólise, dada a possível ocorrência de síndrome HELLP^14^.

Para todas as gestantes sem diagnóstico prévio de DM, independentemente da presença de fatores de risco, é recomendado que a investigação diagnóstica do diabetes gestacional seja feita entre as 24ª-28ª semanas de gestação, através da realização de um teste de tolerância oral à glicose^66^.

A Tabela 4 demonstra as principais medicações utilizadas em pacientes em diálise e a classificação do FDA (*Food and Drug Administration*) em relação à gestação^67^.

**Tabela 4:** Principais medicações utilizadas em pacientes com DRC durante a gestação^67^

Categorias	Drogas	Categorias do FDA
**Medicamentos considerados seguros**		
Agente estimulador da eritropoiese	Eritropoetina	Não atribuído
Álcali	Bicarbonato de sódio	C
Antiagregante plaquetário	Ácido acetilsalicílico	A
Anticoagulante	Heparina de baixo peso molecular	B
Anti-hipertensivos	Alfa-metildopa	B
	Pindolol	B
	Hidroclorotiazida	B
	Clonidina	C
	Metoprolol	C
	Nifedipino	C
Hipouricêmico	Alopurinol	C
Suplemento endovenoso de ferro	Sacarato de hidróxido férrico	B
Suplemento oral de ferro	Sulfato de ferro	Não atribuído
Suplemento de cálcio	Carbonato de cálcio	Não atribuído
Vitamina D	Colecalciferol	Não atribuído
**Medicamentos não seguros**		
Anticoagulante	Warfarina	Não atribuído
	Rivaroxabana/Apixabana	C
Análogos de vitamina D	Calcitriol/Paricalcitol	C
Anti-hipertensivos	Atenolol	D
	Nifedipina de curta duração	X
	Inibidores da enzima conversora de angiotensina	X
	Bloqueadores do receptor da angiotensina	X
Calcimimético	Cinacalcete	C
Quelante de fósforo	Sevelamer	C

FDA: Food and Drug Administration. Categoria A refere-se à evidência de que não há risco para fetos em estudos controlados em humanos; a Categoria B refere-se à ausência de evidência de risco para o feto; a Categoria C refere-se à impossibilidade de excluir risco para o feto; a Categoria D refere-se à evidência positiva de risco para o feto; e a Categoria X refere-se à contraindicação durante a gestação.

### Dieta e Controle Nutricional

O plano nutricional para gestantes em diálise deve respeitar o aumento da necessidade de energia, proteínas, vitaminas e minerais da gestação, de forma a promover o crescimento e desenvolvimento fetais, e harmonizá-lo com as questões próprias da DRC^68^.

Recomenda-se que a necessidade calórica seja calculada a partir da necessidade de 35 Kcal/kg/dia (peso pré-gestacional), acrescida das necessidades energéticas nutricionais relacionadas à gestação, as quais são estimadas em 85 Kcal/dia no primeiro trimestre, 285 Kcal/dia no segundo trimestre e 475 Kcal/dia no terceiro trimestre^69^. Nas gestantes em DP, em que há absorção de glicose existente no dialisato, a ingesta calórica basal deve ser de 25 Kcal/kg/dia acrescida das necessidades específicas gestacionais^70^. Da quantidade total de calorias diárias, recomenda-se que 30–35% sejam provenientes de gorduras e 45–65%, de carboidratos^56^.

Em relação à ingesta proteica, sugere-se 1,5–1,8 g/kg/dia + 10 g/dia, devido à perda de proteína durante o tratamento dialítico intensificado no período gestacional^14^.

Usualmente, o aumento no aporte proteico e calórico é acompanhado de progressivo aumento do peso seco, considerando o ganho esperado de 1–1,5 kg durante o primeiro trimestre e 0,5 kg/semana a partir do segundo trimestre^56^.

A deficiência de micronutrientes (especialmente vitaminas e minerais hidrossolúveis e dialisáveis) pode provocar efeitos sobre a organogênese fetal, sendo recomendada a suplementação de ácido fólico, vitamina C, tiamina, riboflavina, niacina, vitamina B6 e zinco^68^.

A ingesta de sódio e potássio deve ser individualizada levando em consideração as concentrações séricas dos eletrólitos, PA, medicações e estado volêmico^68^.

### Tratamento da Doença Mineral e Óssea

Entre gestantes em diálise, a reposição de cálcio (através de formulação oral ou da solução de diálise) geralmente é necessária para a formação óssea do feto e prevenção de PE. Em relação à suplementação de cálcio, uma revisão sistemática concluiu pela redução de 55% no risco de PE, sendo ainda mais expressiva em mulheres com dieta pobre em cálcio (74%) e naquelas com risco elevado de PE (78%)^71^. Assim, todas as mulheres devem ser orientadas a ter uma dieta rica em cálcio durante a gestação, recomendando-se suplementação para aquelas com risco para PE e/ou dieta pobre em cálcio^71^ (vide tópico Pré-eclâmpsia). O cálcio sérico deve ser monitorado, evitando-se a hipo ou hipercalcemia (risco de hipoparatireoidismo fetal)^25^.

É esperado que, sob esquema de HD intensiva, a paciente gestante tenda a desenvolver hipofosfatemia, sendo por isso indicado o controle semanal do fósforo sérico para eventual reposição (oral, endovenosa ou suplementação na solução de diálise), minimizando os riscos maternos e fetais (cardíacos, neurológicos e de rabdomiólise)^25^. Na incomum eventualidade de ocorrer hiperfosfatemia, as drogas quelantes de escolha são o carbonato de cálcio e o citrato de cálcio, considerando a segurança e demanda aumentada desse elemento na gravidez. Outros quelantes como o sevelamer, carbonato de lantano, acetato de cálcio, e citrato férrico carecem de segurança comprovada na gestação^72^.

No que diz respeito ao hiperparatireoidismo secundário, a meta de paratormônio na gestante em diálise deve seguir o mesmo padrão da população em diálise, mas cabe considerar o risco do manejo com drogas às quais é atribuída limitada segurança (vide Tabela 4)^25^. Os riscos do hiperparatireoidismo não tratado (incomum devido à intensificação da diálise e aumento da oferta de cálcio) podem causar risco ao feto (hipocalcemia, hiperparatireoidismo secundário e raquitismo)^25^.

A gestante em diálise deve suplementar vitamina D3, usualmente na dose entre 800–1000 UI/dia^70^. Não está estabelecido até o presente o nível ideal de vitamina D nas gestantes em diálise^73^. De todo modo, sugere-se o emprego de colecalciferol (formulação mais segura na gestação) para obter níveis séricos acima de 30 ng/mL^25^.

### Tratamento de Anemia

Além dos fatores usuais da anemia (decorrente da DRC e da HD crônica^74^), ocorrem ainda as necessidades do feto em desenvolvimento e a desproporção entre o aumento crescente do volume plasmático materno em relação à produção eritrocitária^75^.

A suplementação de ferro é geralmente necessária principalmente após o segundo trimestre^76^, sendo mais efetiva a alternativa de administração endovenosa, posto que a absorção enteral de ferro é diminuída na DRC^74^. O tratamento da anemia ferropriva em gestantes deve iniciar-se quando a taxa de hemoglobina for <11 g/dL^76^ e as reservas de ferro devem ser ajustadas da forma usual, seguindo as diretrizes vigentes de DRC, mantendo a ferritina >200 mg/dL para pacientes em HD e >100 mg/dL em pacientes em DP, e índice de saturação da transferrina entre 20–30%^74^.

Priorizam-se formulações de ferro seguras na gestação, sendo o sulfato ferroso e o sacarato de hidróxido férrico as formas mais usadas no Brasil. O sulfato ferroso pode ser usado na gestação (categoria A)^77^ e o sacarato de hidróxido férrico pode ser usado pesando risco-benefício, pois, apesar de não haver estudos em gestantes, não tem efeitos adversos fetais em estudos animais (categoria B)^76^. O ferro sucrose tem baixo potencial alergênico, menor tendência ao acúmulo em órgãos viscerais e não tem conservantes tóxicos para a gestação^25,78^. Por outro lado, o gluconato férrico de sódio contém conservantes com álcool benzil, que podem levar a eventos adversos fetais^25^. A carboximaltose férrica (apesar de contar com estudo mostrando segurança^78^) e a derisomaltose férrica não foram aprovadas pelo FDA para uso em gestantes^25^.

Em relação aos AEE, a maioria das gestantes em diálise necessita deles para manter a hemoglobina adequada (conforme diretrizes vigentes de anemia em DRC^74^), com ajuste perfazendo aumento de 50–100% da dose habitual^79^. Como no restante da população em diálise, o emprego de doses de AEE para obter hemoglobina superior ao alvo incrementa o risco de HA e trombose^74^. Uma vez que os AEE não atravessam a barreira hematoplacentária, são considerados seguros na gestação, sendo a epoetina alfa a de segurança obstétrica mais estudada^79^. Estudo com darbepoetina alfa também mostrou segurança na gravidez^79^.

Os inibidores da enzima prolil-hidroxilase do fator induzido por hipóxia, medicamentos orais para anemia em diálise, não têm segurança comprovada para uso em gestantes em diálise^80^.

## Tratamento Dialítico Durante a Gestação

Atualmente, existem evidências suficientes para afirmar que a maior dose de diálise é fundamental para o melhor desfecho fetal, traduzidas na recomendação de que gestantes devem realizar diálise com frequência entre 6-7 sessões semanais, considerando que o aumento da frequência semanal de diálise é fundamental para reduzir o ambiente urêmico materno e fetal^11,43,56^.

Existe, entretanto, grande heterogeneidade dos estudos tratando do número de horas semanais e da monitorização de dose de diálise na paciente gestante^9,10,11,13,81,83^. Os dois grupos de estudo com melhor resultado em relação à sobrevida fetal utilizam estratégias bastante distintas. No Canadá, Hladunewich et al.^82^ baseiam-se no número de horas semanais de HD, sendo preconizado uma dose mínima de 36 horas semanais. Já Baouche et al.^11^ sugerem a utilização de BUN (ureia nitrogenada) < 35 mg/dL (ureia < 75 mg/dL) pré-diálise no meio da semana como alvo para ajuste da dose. Conjuntamente, cabe reconhecer que aumentar a dose de diálise e manter BUN em valores bem próximos ao fisiológico estão associados à maior sobrevida fetal, idade gestacional e peso fetal ao nascer, e menor risco de polihidrâmnio^10,11,18,83,84^.

Assymira et al.^84^ observaram correlação significativa da BUN pré-diálise com o peso fetal e a idade gestacional. No trabalho de Luders et al.^18^, BUN aparece como uma variável independente do desfecho fetal. Observa-se, ainda, que pacientes com BUN pré-diálise <35 mg/dL colhida no meio da semana, ao longo da gestação, apresentaram um risco 6,4 vezes menor de desfecho fetal desfavorável (óbito fetal e/ou feto nascido com menos de 30 semanas de idade gestacional). A associação entre intensidade de diálise e risco de parto prematuro foi analisada por Oliverio et al.^85^, que não foram capazes de observar associação significativa entre dose semanal de diálise ou BUN pré-diálise com risco de parto prematuro ou parto cesáreo.

No entanto, para pacientes que iniciaram diálise em função da gestação, essa dose de diálise bastante elevada não se mostrou benéfica. Ao comparar-se as pacientes que iniciaram diálise já gestando, uma coorte americana realizava 15 ± 4 horas semanais de diálise *versus* 33 ± 6 horas semanais da coorte canadense, tiveram o mesmo desfecho fetal, sugerindo que pacientes que iniciam diálise na gestação não precisam de doses tão elevadas de diálise e que a presença de *clearance* residual deveria nortear a intensidade prescrita de diálise. Sugerem, ainda, a utilização como alvo do valor da ureia pré-diálise de segunda-feira, entre 60–90 mg/dL, nos esquemas de 6 sessões semanais^9^.

O polidrâmnio é relativamente frequente em gestantes em diálise e associa-se ao aumento do risco de parto prematuro, ruptura prematura de membranas e óbito fetal. Explica-se pelo aumento de diurese fetal na presença de níveis mais elevados de ureia (diurese osmótica). Foi demonstrado que o aumento da dose de diálise, com consequente redução dos níveis de ureia materno e fetal, reverte habitualmente essa complicação^18^.

Em DP, há escassez de estudos dedicados à relação entre o aumento da dose de diálise e os desfechos obstétricos, em parte pela ocorrência menos frequente de gestação nessa modalidade (0,25% ao ano)^5,6^. Usualmente, em especial no terceiro trimestre, adota-se otimização da prescrição de DP com emprego de maior tempo de sessão de diálise peritoneal automatizada, e maior número de ciclos com menor volume de infusão a cada ciclo^86,87^. Já na diálise peritoneal ambulatorial contínua, pode-se reduzir o volume de infusão com maior número de trocas. Em metanálise recente, foi evidenciado maior risco de retardo de crescimento fetal nessa modalidade quando comparado com a HD. Esses achados são, no entanto, questionáveis, pois, o grupo DP era composto por relatos de casos ou séries de casos^13^. No geral, os diferentes autores sugerem que as pacientes em diálise, ao engravidarem, mantenham o método dialítico, porém as pacientes iniciando diálise em função da gestação são habitualmente indicadas à HD^43^.

## Conclusão

Ainda que a fertilidade seja reduzida em mulheres em diálise, a gravidez ainda é possível, e as taxas de natalidade aumentam ao longo do tempo. A tomada de decisão compartilhada deve ser o objetivo para o planejamento familiar de todas as mulheres em idade fértil em diálise. A gravidez em diálise continua sendo um cenário clínico desafiador e de alto risco, beneficiando-se da expertise multidisciplinar e interprofissional para fornecer cuidado especializado e aumentar as taxas de natalidade, como tem sido observado em estudos contemporâneos observacionais. Estudos de longo prazo sobre os resultados de gestação em diálise ainda estão em desenvolvimento, mas o crescente corpo de literatura vem contribuindo com as recomendações com que aconselhamos nossas pacientes. Em especial, a diálise intensificada é recomendada para gestantes em diálise, associada a melhores resultados, incluindo aumento da idade gestacional e peso ao nascer.
